# Comparison of efficacy and safety between robotic-assisted versus laparoscopic surgery for locally advanced mid-low rectal cancer following neoadjuvant chemoradiotherapy: a systematic review and meta-analysis

**DOI:** 10.1097/JS9.0000000000001854

**Published:** 2024-06-24

**Authors:** Xin-Mao Zhu, Xiao Bai, Hai-Qi Wang, Dong-Qiu Dai

**Affiliations:** a Department of Surgical Oncology, The Fourth Affiliated Hospital of China Medical University; b Cancer Center, The Fourth Affiliated Hospital of China Medical University, Shenyang, People’s Republic of China

**Keywords:** laparoscopic, meta-analysis, neoadjuvant therapy, rectal cancer, robotic

## Abstract

**Background:**

To some extent, the robotic technique does offer certain benefits in rectal cancer surgery than laparoscopic one, while remains a topic of ongoing debate for rectal cancer patients who have undergone neoadjuvant chemoradiotherapy (NCRT).

**Methods:**

Potential studies published until January 2024 were obtained from Web of Science, Cochrane Library, Embase, and PubMed. Dichotomous and continuous variables were expressed as odds ratios (ORs) or weighted mean differences (WMDs) with 95% CIs, respectively. A random effects model was used if the *I* ^2^ statistic >50%; otherwise, a fixed effects model was used.

**Results:**

Eleven studies involving 1079 patients were analysed. The robotic-assisted group had an 0.4 cm shorter distance from the anal verge (95% CI: −0.680 to −0.114, *P*=0.006) and 1.94 times higher complete total mesorectal excision (TME) rate (OR=1.936, 95% CI: 1.061–3.532, *P*=0.031). However, the operation time in the robotic-assisted group was 54 min longer (95% CI: 20.489–87.037, *P*=0.002) than the laparoscopic group. In addition, the robotic-assisted group had a lower open conversion rate (OR=0.324, 95% CI: 0.129–0.816, *P*=0.017) and a shorter length of hospital stay (WMD=−1.127, 95% CI: −2.071 to −0.184, *P*=0.019).

**Conclusion:**

Robot-assisted surgery offered several advantages over laparoscopic surgery for locally advanced mid-low rectal cancer following NCRT in terms of resection of lower tumours with improved TME completeness, lower open conversion rate, and shorter hospital stay, despite the longer operative time.

## Introduction

Highlights

- A meta-analysis was conducted to compare robotic-assisted surgery and laparoscopic surgery for locally advanced mid-low rectal cancer after neoadjuvant chemoradiotherapy.
- The review included 11 studies with 1079 patients.
- Robot-assisted surgery offered several advantages over laparoscopic surgery for locally advanced mid-low rectal cancer following neoadjuvant chemoradiotherapy in terms of resection of lower tumours with improved total mesorectal excision completeness, lower open conversion rate, and shorter hospital stay, despite the longer operative time.
- Compared to conventional laparoscopic surgery, robot-assisted surgery showed similar pathological outcomes, postoperative complications, and oncological outcomes.

Despite an overall decline in the incidence of colorectal cancer, the proportion of rectal cancer is gradually increasing, with diagnoses occurring at a younger age and tumours at a later stage^1^. Total mesorectal excision (TME) has become the standard procedure for the treatment of rectal cancer due to its ability to reduce recurrence rates and prolong survival^2^. Additionally, neoadjuvant chemoradiotherapy (NCRT) followed by TME, with or without adjuvant chemotherapy, could reduce the recurrence rate and increase the rate of sphincter preservation of locally advanced mid-low rectal cancer^3^.

In recent years, minimally invasive techniques have undergone continuous technological advances and achieved oncological outcomes comparable to those of open surgery, with less blood loss, as well as a lower rate of postoperative complications and a faster postoperative recovery^4–6^. However, laparoscopic surgery has limitations, including restricted visibility, less flexible instrument manipulation, and unstable camera images. Robotic-assisted surgery overcomes these deficiencies and is particularly suitable for patients who are challenging to operate on with laparoscopic surgery, such as those with mid-low rectal cancer or gynaecological tumours. Dating back to 2002, the first report on robotic surgery for colorectal cancer was published^7^. Subsequently, robot-assisted surgery has been used in a variety of pelvic surgeries and has become increasingly popular among surgeons^8,9^.

Previous studies have shown comparable short-term and long-term outcomes, but robotic surgery offers the additional benefits of lower open conversion rate and faster recovery when comparing robotic and laparoscopic approaches for mid-low rectal cancer. However, it is important to note that not all studies included patients who had undergone NCRT^10–16^. In 2013, Saklani *et al*.^17^ first reported the inclusion of patients after NCRT, showing comparable oncological efficacy between the two surgical approaches. Robotic surgery has been reported advantages in certain aspects, such as a higher number of lymph nodes retrieved^18^ and lower anastomotic leakage and urinary retention rates^19^. However, robotic surgery costs more than laparoscopic surgery^20,21^. Therefore, robotic surgery for this issue remains ongoing debate.

To our knowledge, there is no meta-analysis offering the superior surgical approach after NCRT in locally advanced mid-low rectal cancer. This study therefore evaluated the effectiveness and safety of the robotic and laparoscopic surgery by systematically comparing the intraoperative parameters, pathological results, postoperative complications, and oncological outcomes.

## Materials and methods

We did this research in compliance with the Preferred Reporting Items of Systematic Reviews and Meta-Analyses (PRISMA, Supplemental Digital Content 1, http://links.lww.com/JS9/C856, Supplemental Digital Content 2, http://links.lww.com/JS9/C857) guidelines^22^. It was registered on PROSPERO, which provides more detailed information. Each quality assessment was performed according to AMSTAR 2 (Supplemental Digital Content 3, http://links.lww.com/JS9/C858)^23^.

### Search strategy

Web of Science, Cochrane Library, Embase, and PubMed were systematically searched to retrieve potential studies up to January 2024 by two authors. The literature was searched using the following keywords: ‘neoadjuvant therapy’, ‘rectal neoplasms’, ‘robotic surgery’, ‘laparoscopy’, and their variants. Furthermore, eligible studies were manually checked in the reference lists of the included articles. The search strategies presented are shown in Supplementary Table S1 (Supplemental Digital Content 4, http://links.lww.com/JS9/C859).

### Study outcomes

Study outcomes included (1) intraoperative and postoperative parameters, (2) postoperative complications, (3) pathological outcomes, and (4) oncological outcomes: local recurrence rate, disease-free survival (DFS), and overall survival (OS).

### Inclusion and exclusion criteria

The PICOS criteria were met: (1) P (patients): patients with locally advanced mid-low rectal cancer after NCRT; (2) I (intervention): robotic-assisted rectal resection; (3) C (comparison): laparoscopic rectal resection; (4) O (outcomes): at least one study outcome; (5) S (study design): there was no restriction on study type, which meant that a randomised controlled trial or nonrandomised controlled trial (NRCT) was acceptable. Furthermore, articles from any country must be in English.

Exclusion criteria were: (1) reviews, meta-analyses, case series, commentaries, case reports, correspondence with authors or editors, editorials, surgical technical notes, and conference abstracts; (2) studies without a control group; (3) inability to access the full text; (4) articles with duplicate data.

### Data extraction and quality assessment

Two authors independently extracted the data and carried out the quality assessment, any disagreements were resolved by third-party arbitration. If there were any disagreements during the data extraction process, the two authors resolved them through discussion or sought the assistance of a third author for arbitration. The following data and study outcomes were extracted: country, publication year, name of the first author, sample size, study design and period, baseline characteristics of patients, intraoperative and postoperative parameters, postoperative complications (including overall postoperative complications, Clavien–Dindo grade I or II complications, III or higher complications, anastomosis leakage, bleeding, postoperative ileus, bleeding, urine retention, urinary dysfunction, urinary infection, intra-abdominal infection, surgical site infection, pulmonary complication, and lymphatic leakage), pathological outcomes (including tumour size, harvested number of lymph nodes, positive circumferential resection margin [CRM], and distal resection margin), and long-term oncological outcomes. Microsoft Excel was used to record information about the included studies. If data on primary outcomes were not available, it was the intention to contact the corresponding author of the original study.

The Newcastle–Ottawa Scale (NOS) was used to judge the quality of NRCT, which consists of three components: selection of patients, comparability between groups, and outcome or exposure. Each article could score a maximum of 9 points, which was then categorised as low (0–3), moderate (4–6), or high (7–9) quality^24^.

### Statistical analysis

Dichotomous and continuous variables were expressed as odds ratios (ORs) and weighted mean differences (WMDs) with 95% CIs, respectively. Where continuous outcomes were reported in the literature as medians, ranges, and interquartile ranges, these were converted to means and SD using the guidelines in the Cochrane Handbook for Statistical Analysis or the formula described by Shi *et al*.^25^. Heterogeneity among studies was assessed using the *χ* ^2^ test (Cochrane *Q* test) and the *I* ^2^ statistic. *I*² <25%, 25–50%, and >50% were considered as low, moderate, and high heterogeneity, respectively. If *P*<0.10 or *I* ^2^ >50%, a random-effects model was used for the analysis; otherwise, a fixed-effects model was used.

Subgroup analyses based on region, study design, and the interval between surgery and NCRT were performed when the *I* ^2^ statistic for the outcome of interest was >50%. Statistical significance was considered when the *P*-value was <0.05 (two-sided). Funnel plots, Begg’s^26^, and Egger’s test^27^ were generated or applied to assess publication bias. If publication bias was detected, the trim-and-fill method was applied to identify unpublished studies. To assess the stability of the results, a sensitivity analysis was performed. All statistical analysis was conducted using Stata 17.0 and Review Manager 5.4.

## Results

**Figure 1:**
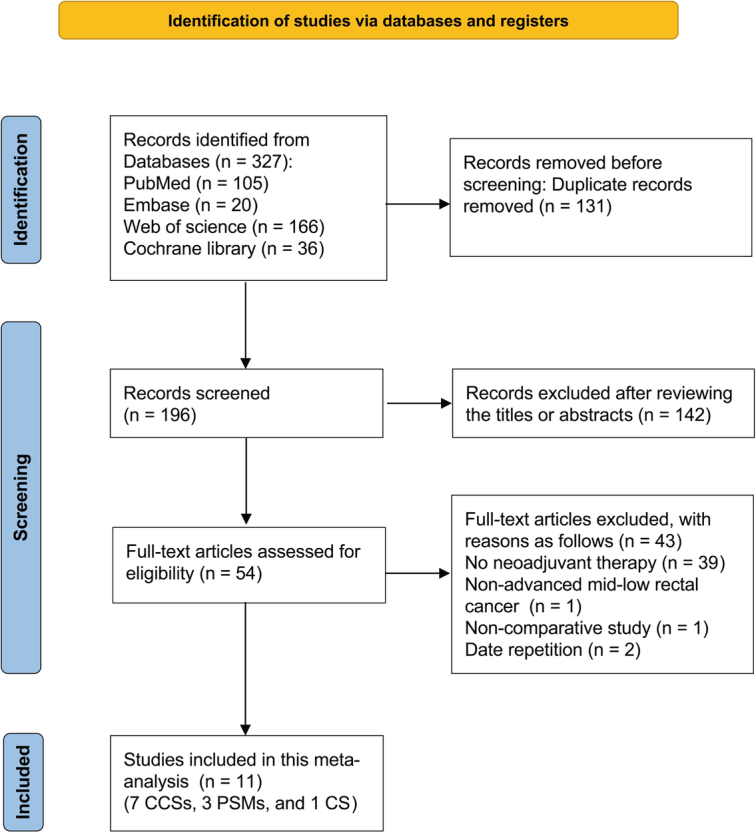
Flowchart for study retrieval. CCS, case–control study; CS, cohort study; PSM, propensity score-matched study.

The search yielded 327 articles, of which 131 duplicates were removed, leaving 196 articles. After reviewing the titles and abstracts, 142 articles were excluded. Finally, based on the inclusion and exclusion criteria, 11 studies published between 2016 and 2023 were included^19,28–37^. The literature selection was detailed in Figure 1.

### Characteristics of included studies

These studies included a total of 1079 patients, 519 (48.1%) in the robotic-assisted group and 560 (51.9%) in the laparoscopic group. The 11 eligible studies were conducted in China (3), Korea (3), Switzerland (1), Turkey (1), and Japan (3), and their baseline characteristics are presented in Table 1. All studies included in this research were NRCTs. The quality assessment was shown in Supplementary Table S2 (Supplemental Digital Content 4, http://links.lww.com/JS9/C859). The results indicate that all studies were of high quality, with a NOS score of 7 or higher.

**Table 1:** Characteristics of included studies in this meta-analysis.

										ASA score, *n*			
Study	Country	Study design	Study period	Group	Simple size	Operation interval (weeks)	Age (y)	Male/Female	BMI (kg/m^2^)	I	II	III	Distance from anal verge (cm)
Zhang L *et al*. 2023^28^	China	CCS	2015.1–2021.4	Robotic	37	6-12	58.1±12.8	11/26	21.7±3.4	33		4	4.2±2.0
				Laparoscopic	44		57.6±7.6	21/23	23.4±2.7	37		7	5.5±2.1
Yamanashi T *et al*. 2023^19^	Japan	PSM	2014.2–2022.2	Robotic	30	8-10	62.4±11.0	24/6	22.4±2.7	7	21	2	4.61±1.64
				Laparoscopic	30		61.7±10.3	26/4	22.7±3.1	7	18	5	4.74±1.65
Lim S *et al*. 2023^29^	Japan	CCS	2005–2020	Robotic	46	6-8	61±11.4	29/17	22.6±3.0	NA			5.54±1.4
				Laparoscopic	64		63±10.4	43/21	22.4±3.3	NA			5.98±1.61
Ishizaki T *et al*. 2023^30^	Japan	CCS	2013.1–2022.7	Robotic	27	3-4	61.0±9.5	20/7	21.6±3.7	11	16	0	NA
				Laparoscopic	33		57.5±13.7	22/11	23.0±3.7	14	18	1	NA
Piozzi GN *et al*. 2022^31^	Korea	CCS	2006.9–2019.9	Robotic	124	8-10	55.7±13.5	86/38	23.7±2.9	22	98	4	6.04±2.93
				Laparoscopic	36		60.0±11.2	28/8	23.2±3.6	13	22	1	6.15±3.09
Chen TC *et al*. 2022^32^	China	PSM	2011.11–2018.12	Robotic	56	6-8	57.4±11.2	39/17	23.5±3.6	0	32	24	4.464±1.991
				Laparoscopic	56		56.3±11.0	38/18	23.5±3.2	0	28	28	4.214±1.933
Angehrn FV *et al*. 2022^33^	Switzerland	CS	2015.3–2020.6	Robotic	38	NA	67.4±16.9	29/9	24.9±3.9	0	26	12	8.50±3.85
				Laparoscopic	64		63.7±12.1	42/22	25.6±3.3	0	42	22	8.65±2.28
Asoglu O *et al*. 2020^34^	Turkey	CCS	2005.1–2013.12	Robotic	14	NA	55.5±24.7	14/0	24.9±3.3	NA			NA
				Laparoscopic	65		54.9±39.4	65/0	26.4±8.3	NA			NA
Lim DR *et al*. 2017^35^	Korea	CCS	2006.1–2010.12	Robotic	74	6-8	65.1±12.4	50/24	23.4±2.9	50	24	0	5.3±2.3
				Laparoscopic	64		65.8±11.1	46/18	22.7±2.9	33	29	2	6.7±2.6
Huang YM *et al*. 2017^36^	China	CCS	2012.1–2015.4	Robotic	40	6-8	60.0±12.2	25/15	23.0±4.4	36		4	6.8 ±3.2
				Laparoscopic	38		60.1±14.2	28/10	24.3±3.5	31		7	6.3±3.4
Kim YS *et al*. 2016^37^	Korea	PSM	2010.3–2012.1	Robotic	33	4-8	57.0±9.6	23/10	23.2±2.3	15	18	0	5.41±1.9
				Laparoscopic	66		58.2±9.8	46/20	23.3±3.1	37	25	4	5.57±2.1

ASA, American Society of Anesthesiologists Physical Status Classification; CCS, case-control study; CS, cohort study; NA, not available; PSM, propensity score-matched study.

### Patient characteristics

**Figure 2:**
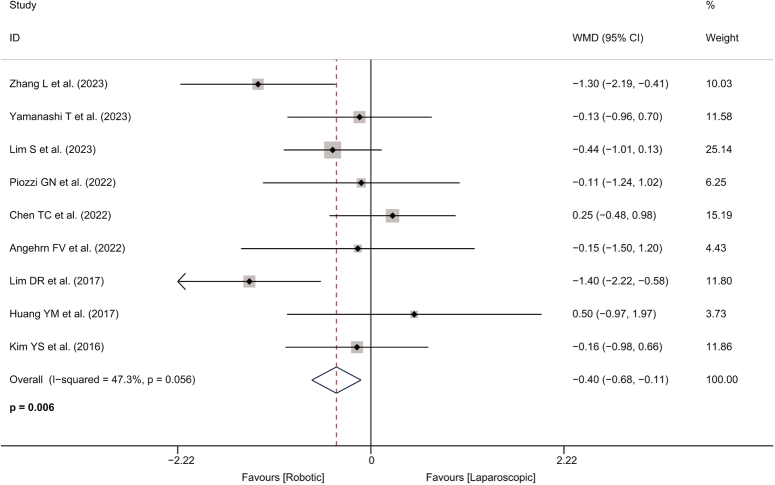
Forest plot of distance from anal verge in the robotic and laparoscopic groups. WMD, weighted mean difference.

No significant differences in age (WMD=−0.323; 95% CI: −1.792 to 1.145; *P*=0.666; *I* ^2^=0.0%), male sex (OR=0.856, 95% CI: 0.647–1.134, *P*=0.278; *I* ^2^=0.0%), BMI (WMD=−0.215, 95% CI: −0.621 to 0.191, *P*=0.299; *I* ^2^=29.4%), and previous abdominal surgery (OR=0.654, 95% CI: 0.407–1.052, *P*=0.080; *I* ^2^=0.0%) between two groups were observed. However, the distance from anal verge in the robotic-assisted group was about 0.4 cm shorter than in the laparoscopic group (WMD=−0.397, 95% CI: −0.680 to −0.114, *P*=0.006; *I* ^2^=47.3%; Fig. 2). No significant heterogeneity was observed for any of the outcomes listed above. Table 2 shows a pooled analysis of patient characteristics.

**Table 2:** Meta-analysis of endpoints of interest.

					Heterogeneity	
Outcomes	Number of studies	Number of patients	Effect size (95% CIs)	*P*	*I* ^2^ statistic (%)	*P*
Characteristics						
Age	11	1079	WMD: −0.323 ( −1.792, 1.145)	0.666	0.0	0.625
Male sex	11	1079	OR: 0.856 (0.647–1.134)	0.278	0.0	0.711
BMI (kg/m^2^)	11	1079	WMD: −0.215 ( −0.621, 0.191)	0.299	29.4	0.166
Distance from anal verge, cm	9	940	WMD: −0.397 ( −0.680, −0.114)	0.006	47.3	0.056
Previous abdominal surgery	5	530	OR: 0.654 (0.407–1.052)	0.080	0.0	0.978
Intraoperative and postoperative parameters						
Complete TME	5	578	OR: 1.936 (1.061–3.532)	0.031	32.1	0.208
Incomplete TME	2	239	OR: 0.401 (0.174–0.926)	0.032	0.0	0.364
Operation time, minutes	9	959	WMD: 53.763 (20.489–87.037)	0.002	88.7	< 0.001
Conversion to open	6	638	WMD: 0.324 (0.129–0.816)	0.017	38.5	0.149
Hospital stay, days	6	689	WMD: −1.127 ( −2.071, −0.184)	0.019	16.4	0.308
Pathological outcomes						
Tumour size, cm	7	626	WMD: −0.066 ( −0.322, 0.190)	0.614	37.2	0.144
Distal margin, cm	5	485	WMD: −0.003 ( −0.386, 0.381)	0.989	58.5	0.047
Positive CRM	6	689	OR: 1.059 (0.487–2.304)	0.884	2.0	0.403
Harvested No. of lymph nodes	7	781	WMD: 0.790 ( −1.894, 3.474)	0.564	80.9	<0.001
Postoperative complication						
Overall complication	10	1019	OR: 1.037 (0.774–1.387)	0.809	32.0	0.152
Complication Grade <III	4	403	OR: 0.741 (0.453–1.211)	0.231	48.9	0.118
Complication Grade ≥III	6	653	OR: 1.040 (0.619–1.746）	0.883	42.0	0.125
Anastomosis leakage	9	868	OR: 0.657 (0.386–1.119)	0.122	0.0	0.528
Bleeding	3	321	OR: 0.849 (0.165–4.359)	0.845	0.0	0.522
Postoperative ileus	9	868	OR: 1.163 (0.666–2.031)	0.596	6.9	0.377
Urine retention	4	358	OR: 0.477 (0.188–1.211)	0.119	0.0	0.674
Urinary dysfunction	3	248	OR: 1.425 (0.456–4.448)	0.542	0.0	0.708
Urinary infection	3	262	OR: 0.365 (0.077–1.721)	0.203	0.0	0.453
Intra-abdominal infection	5	436	OR: 0.605 (0.178–2.051)	0.419	0.0	0.731
Surgical site infection	6	528	OR: 0.708 (0.288–1.738)	0.451	0.0	0.815
Pulmonary complication	2	183	OR: 1.564 (0.337–7.257)	0.568	0.0	0.534
Lymphatic leakage	2	198	OR: 1.760 (0.221–13.989)	0.593	0.0	0.727
Oncological outcomes						
3-OS	3	379	OR: 0.842 (0.440–1.612)	0.604	0.0	0.952
5-OS	4	489	OR: 0.806 (0.476–1.366)	0.423	0.0	0.855
3-DFS	2	219	OR: 0.975 (0.520–1.830)	0.938	0.0	0.880
5-DFS	3	329	OR: 1.446 (0.858–2.437)	0.166	0.0	0.429
Local recurrence	6	670	OR: 1.165 (0.574–2.364)	0.672	0.0	0.526

CRM, circumferential resection margin; DFS, disease-free survival; OR, odds ratio; OS, overall survival; TME, total mesorectal excision; WMD, weighted mean difference.

### Intraoperative and postoperative parameters

**Figure 3:**
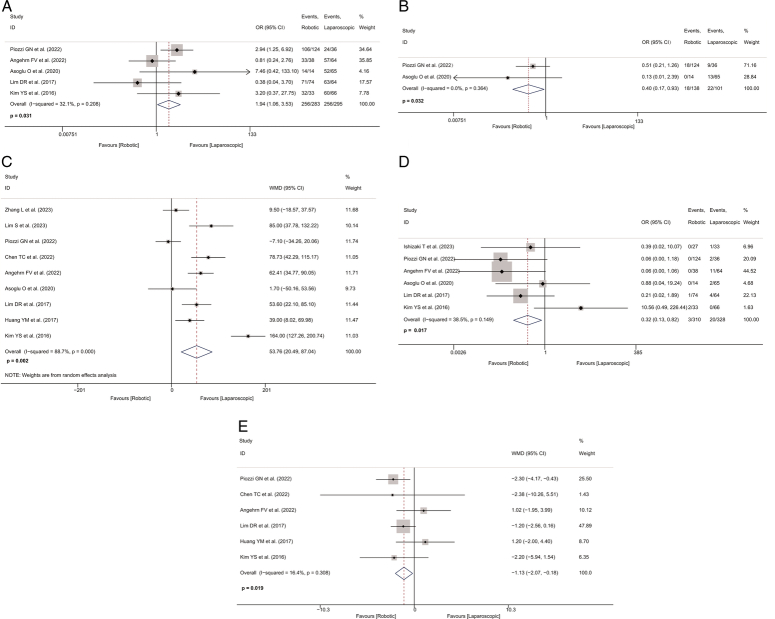
Forest plot of intraoperative and postoperative parameters in the robotic and laparoscopic groups. A, Forest plot of complete total mesorectal excision rate in the robotic and laparoscopic groups. B, Forest plot of incomplete total mesorectal excision rate in the robotic and laparoscopic groups. C, Forest plot of operative time in the robotic and laparoscopic groups. D, Forest plot of conversion to open surgery rate in the robotic and laparoscopic groups. E, Forest plot of length of stay in the robotic and laparoscopic groups. OR, odds ratio; WMD, weighted mean difference.

Data on complete TME were available from five studies^31,33–35,37^. The incidence of complete TME in the robotic-assisted group was ~1.94 times higher than that in the laparoscopic group (OR=1.936, 95% CI: 1.061–3.532, *P*=0.031; *I* ^2^=32.1%; Fig. 3A).

Two studies with 239 patients provided data on incomplete TME^31,34^. The robotic-assisted group had a lower incidence of incomplete TME (OR=0.401, 95% CI: 0.174–0.926, *P*=0.032; *I* ^2^=0.0%; Fig. 3B).

Nine studies provided data on operative time, with a total of 959 patients included in this meta-analysis^28,29,31–37^. Compared to the laparoscopic group, operative time was about 54 min longer in the robotic-assisted group (WMD=53.763, 95% CI: 20.489–87.037, *P*=0.002; *I* ^2^=88.7%; Fig. 3C). However, there was high heterogeneity in operative time.

Data on conversion to open surgery were available from six studies, with 638 patients included in this meta-analysis^30,31,33–35,37^. The pooled analysis indicated that the robotic-assisted group had a lower incidence of conversion to open surgery compared to the laparoscopic group (OR=0.324, 95% CI: 0.129–0.816, *P*=0.017; *I* ^2^=38.5%; Fig. 3D).

Length of hospital stay was reported in six studies with a total of 689 patients^31–33,35–37^. The length of hospital stay in the robotic-assisted group was ~1 day shorter than in the laparoscopic group (WMD=−1.127, 95% CI: −2.071 to −0.184, *P*=0.019; *I* ^2^=16.4%; Fig. 3E). Detailed pooled results of intraoperative and postoperative parameters are shown in Table 2.

### Pathological outcomes

**Figure 4:**
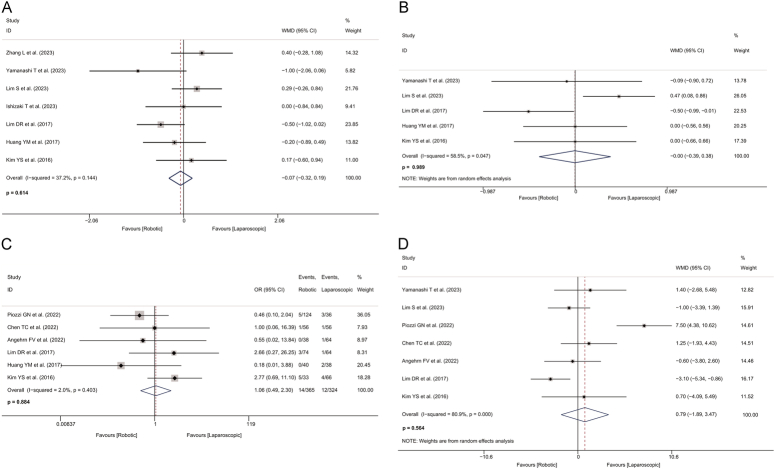
Forest plot of pathological outcomes in the robotic and laparoscopic groups. A, Forest plot of tumour size in the robotic and laparoscopic groups. B, Forest plot of distal resection margin in the robotic and laparoscopic groups. C, Forest plot of positive circumferential resection margin in the robotic and laparoscopic groups. D, Forest plot of the total number of lymph nodes harvested in the robotic and laparoscopic groups. OR, odds ratio; WMD, weighted mean difference.

The results indicated that no significant differences were observed between the two groups in the following parameters: tumour size (WMD=−0.066, 95% CI: −0.322 to 0.190, *P*=0.614; *I* ^2^=37.2%; Fig. 4A), distal resection margin (WMD=−0.003, 95% CI: −0.386 to 0.381, *P*=0.989; *I* ^2^=58.5%; Fig. 4B), positive CRM (OR=1.059, 95% CI: 0.487–2.304, *P*=0.884; *I* ^2^=2%; Fig. 4C), and number of harvested lymph nodes (WMD=0.790, 95% CI: −1.894 to 3.474, *P*=0.564; *I* ^2^=80.9%; Fig. 4D) between the robotic-assisted and the laparoscopic groups. However, there was significant heterogeneity in the distal resection margin and number of harvested lymph nodes. Detailed pooled results for the above pathological outcomes are shown in Table 2.

### Postoperative complications

#### Overall postoperative complications

**Figure 5:**
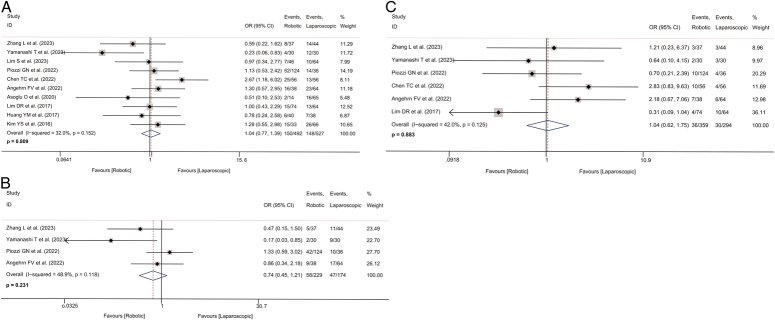
Forest plot of postoperative complications in the robotic and laparoscopic groups. A, Forest plot of overall postoperative complications in the robotic and laparoscopic groups. B, Forest plot of Clavien–Dindo grade I or II complications in the robotic and laparoscopic groups. C, Forest plot of Clavien–Dindo grade III or IV complications in the robotic and laparoscopic groups. OR, odds ratio.

Ten studies provided data on overall postoperative complications, with a total of 1019 patients included in this meta-analysis^19,28,29,31–37^. The pooled analysis showed that there was no statistically significant difference in overall postoperative complications between the robotic-assisted and the laparoscopic groups (OR=1.037, 95% CI: 0.774–1.387, *P*=0.809; *I* ^2^=32.0%; Table 2; Fig. 5A). The heterogeneity was moderate.

#### Clavien–Dindo grade I or II complications

Data on Clavien–Dindo grade I or II complications were available from 4 studies, with 403 patients included in this meta-analysis^19,28,31,33^. The difference in Clavien–Dindo grade I or II complications between the robotic-assisted and the laparoscopic groups was not statistically significant (OR=0.741, 95% CI: 0.453–1.211, *P*=0.231; *I* ^2^=48.9%; Table 2; Fig. 5B). Additionally, there was moderate heterogeneity.

#### Clavien–Dindo grade III or IV complications

Six studies reported Clavien–Dindo grade III or IV complications, totalling 653 patients^19,28,31–33,35^. Clavien–Dindo grade III or IV complications were not statistically significantly different between the two groups (OR=1.040, 95% CI: 0.619–1.746, *P*=0.883; *I* ^2^=42.0%; Table 2; Fig. 5C). There was no significant heterogeneity between groups.

#### Detailed complications

Pooled analyses showed that no statistically significant differences were observed between the robotic-assisted and the laparoscopic groups in the following complications: anastomosis leakage, postoperative bleeding, postoperative ileus, urine retention, urinary dysfunction, urinary infection, intra-abdominal infection, surgical site infection, pulmonary complication, and lymphatic leakage. Heterogeneity was low for all of the outcomes compared. The pooled analyses of postoperative complications are detailed in Table 2. The forest plots for the various complications are shown in Supplementary Figure S1-S10 (Supplemental Digital Content 5, http://links.lww.com/JS9/C860), respectively.

### Oncological outcomes

#### OS

**Figure 6:**
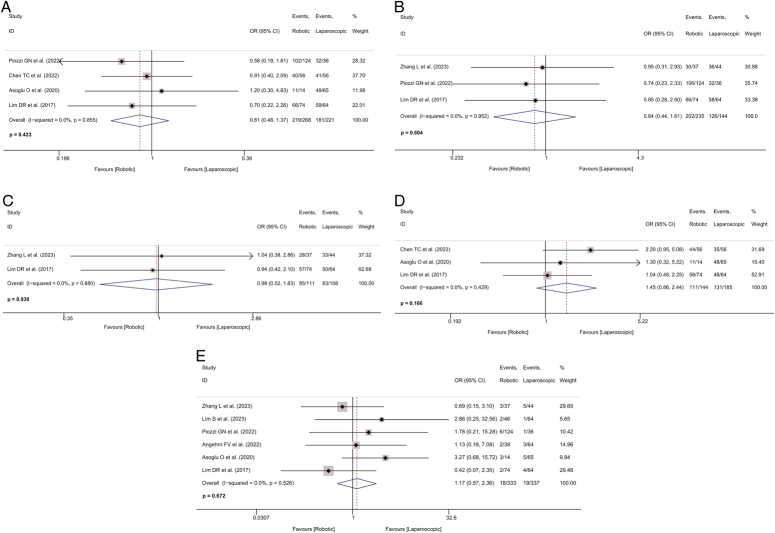
Forest plot of oncological outcomes in the robotic and laparoscopic groups. A, Forest plot of 5-year overall survival in the robotic and laparoscopic groups. B, Forest plot of 3-year overall survival in the robotic and laparoscopic groups. C, Forest plot of 3-year disease-free survival in the robotic and laparoscopic groups. D, Forest plot of 5-year disease-free survival in the robotic and laparoscopic groups. E, Forest plot of local recurrence rate in the robotic and laparoscopic groups. OR, odds ratio.

Data on 5-year OS was available from four studies^31,32,34,35^, and 3-year OS was assessed from three studies^28,31,35^. No significant difference was found between the robotic-assisted and laparoscopic groups in 5-year and 3-year OS (OR=0.806, 95% CI: 0.476–1.366, *P*=0.423; *I* ^2^=0.0%; Fig. 6A; OR=0.842, 95% CI: 0.440–1.612, *P*=0.604; *I* ^2^=0.0%; Fig. 6B; respectively; Table 2). There was no significant heterogeneity.

#### DFS

Two studies^28,35^ and three studies^32,34,35^ reported data on 3-year and 5-year DFS, with a total of 219 and 329 patients, respectively. There was also no significant difference in 3-year and 5-year DFS between the two groups (OR=0.975, 95% CI: 0.520–1.830, *P*=0.938; *I* ^2^=0.0%; Fig. 6C; OR=1.446, 95% CI: 0.858–2.437, *P*=0.166; *I* ^2^=0.0%; Fig. 6D; respectively; Table 2). There was no significant heterogeneity between the groups.

#### Local recurrence rate

Local recurrence rate was reported in six studies, with 670 patients included in this meta-analysis^19,29,31,33–35^. No significant difference was observed between the two groups in local recurrence rate in follow-up periods (OR=1.165, 95% CI: 0.574–2.364, *P*=0.672; *I* ^2^=0.0%; Table 2; Fig. 6E). No significant heterogeneity existed.

### Subgroup analysis

Subgroup analysis was performed for outcomes with significant heterogeneity (operative time, distal resection margin, and number of lymph nodes harvested). Subgroup analysis was stratified by study design, region, and time of surgery (Table 3).

**Table 3:** Subgroup analysis of the outcomes with significant heterogeneity.

				Heterogeneity	
Subgroup	Number of studies	Effect size (95% CIs)	*P*	*I* ^2^ statistic (%)	*P*
Total number of harvested lymph nodes					
Total	7	WMD: 0.790 (−1.894, 3.474)	0.564	80.9	<0.001
Study design					
PSM	3	WMD: 1.176 (−1.045, 3.397)	0.299	0.0	0.975
CCS	3	WMD: 1.038 (−4.695, 6.770)	0.723	93.4	<0.001
CS	1	WMD:−0.600 (−3.804, 2.604)	0.714	_	_
Region					
Asian	6	WMD: 1.043 (−2.108, 4.193)	0.517	83.9	<0.001
Western	1	WMD:−0.600 (−3.804, 2.604)	0.714	_	_
Timing of surgery					
>8 weeks	2	WMD: 4.597 (−1.374, 10.568)	0.131	81.5	0.020
<8 weeks	4	WMD:−0.936 (−2.955, 1.083)	0.364	47.2	0.128
Operation time					
Total	9	WMD: 53.763 (20.489–87.037)	0.002	88.7	<0.001
Study design					
PSM	2	WMD: 121.332 (37.771–204.893)	0.004	90.4	0.001
CCS	6	WMD: 28.782 (2.658–54.905)	0.031	71.4	0.004
CS	1	WMD: 62.410 (34.770–90.050)	0.001	_	_
Region					
Asian	7	WMD: 59.191 (17.633–100.748)	0.005	91.0	<0.001
Western	2	WMD: 36.183 (−22.759, 95.125)	0.229	75.6	0.043
Timing of surgery					
>8 weeks	1	WMD:−7.100 (−34.260, 20.060)	0.608	_	_
<8 weeks	5	WMD: 83.423 (39.859–126.986)	0.001	86.4	<0.001
Distal resection margin					
Total	5	WMD:−0.003 (−0.386, 0.381)	0.989	58.5	0.047
Study design					
PSM	2	WMD:−0.036 (−0.545, 0.473)	0.890	0.0	0.865
CCS	3	WMD: 0.004 (−0.590, 0.598)	0.989	78.9	0.009
Timing of surgery					
>8 weeks	1	WMD:−0.090 (−0.897, 0.717)	0.827	_	_
<8 weeks	4	WMD: 0.008 (−0.447, 0.462)	0.974	68.5	0.023

CCS, case-control study; CS, cohort study; PSM, propensity score-matched study; WMD, weighted mean difference.

Subgroup analyses of operative time showed that the robotic-assisted group had longer operative times in all subgroups except in the Western subgroup, however, both groups showed significant heterogeneity across subgroups. The subgroup analyses of distal resection margins suggested that their heterogeneity may have been influenced by the study design, and the difference was not statistically significant in all subgroups. Subgroup analysis results indicated that study design and timing of surgery may contribute to high heterogeneity in the number of lymph nodes harvested, and there was no significant difference in all subgroups (Table 3; Supplementary Figures S11–S13, Supplemental Digital Content 5, http://links.lww.com/JS9/C860).

### Publication bias

Funnel plots were used to assess the presence of publication bias for the total number of lymph nodes harvested. The funnel plot appeared to be approximately symmetric, suggesting no significant publication bias (Supplementary Figure S14, Supplemental Digital Content 5, http://links.lww.com/JS9/C860). Additionally, the results of Egger’s test (*P*=0.288) and Begg’s test (*P*=0.548) suggest that there is no significant publication bias (Supplementary Figure S15, Supplemental Digital Content 5, http://links.lww.com/JS9/C860).

### Sensitivity analysis

A sensitivity analysis was conducted for the outcomes with a high heterogeneity (operative time, distal resection margin, and harvested number of lymph nodes). The sensitivity analysis of the total number of lymph nodes harvested is shown in Supplementary Figure S16 (Supplemental Digital Content 5, http://links.lww.com/JS9/C860). No significant changes in the pooled results were observed after each study was removed individually, confirming the stability of the results. Besides, sensitivity analysis results for the operative time and distal resection margins also showed that they were all statistically stable (Supplementary Figures S17-S18, Supplemental Digital Content 5, http://links.lww.com/JS9/C860).

## Discussion

Nowadays, the studies included in the previous meta-analyses did not all include patients who received neoadjuvant treatment^11,13,38,39^. NCRT plays a crucial role in treating locally advanced mid-low rectal cancer by reducing tumour staging, improving surgical options, and enhancing survival. However, patients who receive neoadjuvant treatment may face greater surgical difficulties due to factors such as tissue oedema, fibrosis, invasion of pelvic nerves by enlarged lymph nodes, and pelvic stenosis, which may lead to more postoperative complications or adverse events. Therefore, comparing the effectiveness of robotic and laparoscopic surgery in treating rectal cancer patients after NCRT is essential. Our pooled analysis shows that although the robotic-assisted group had a 54 min longer operative time, they had the ability to remove tumours lower down with a higher TME completeness. Moreover, patients undergoing robotic-assisted surgery had a lower open conversion rate and a shorter duration of hospitalisation.

This study reveals that the robotic-assisted group has a shorter distance from the anal verge with a higher complete TME rate than the laparoscopic group. This difference may be due to the clearer 3D imaging system and more stable robotic surgical procedures, which are advantageous when operating in the narrow pelvic cavity, making it suitable for patients with ultralow rectal cancer^40,41^. Additionally, the robotic-assisted group has a lower open conversion rate and duration of hospitalisation. The main reasons are that robotic surgery addresses the restrictions of traditional laparoscopic techniques, such as a clearer imaging system and more stable robotic surgical arm operations, making it easier to identify tissues such as lymph nodes, blood vessels, and nerves in the narrow pelvic cavity^40,41^. Our meta-analysis results show that the surgical time in the robotic-assisted group was ~54 min longer, which is consistent with the results of meta-analyses published by Wang *et al*.^42^. and Gavriilidis *et al*.^43^. Extended robot docking time, lack of tactile feedback, and lack of experience with robotic surgery may be contributing factors^44–46^. Additionally, in patients with mid-low rectal cancer after NCRT, increased tissue oedema and fibrosis contribute to the formation of smoke and the discharge of fluids during surgery^47^. By improving the robotic surgical system or increasing the experience of the surgical team, the duration of robotic surgery may be reduced^45,46,48^.

Moreover, in terms of one of the most crucial parameters for assessing the short-term efficacy of surgical treatment, previous meta-analyses have shown that robotic rectal cancer surgery is comparable to laparoscopic surgery in terms of early postoperative complications^49,50^. However, some studies suggested that robotic surgery may reduce the risk of urinary retention or alleviate urinary system symptoms, as there is a higher risk of damage to the sacral nerves and the parasympathetic nervous system with traditional laparoscopic surgery^51,52^. Meta-analysis results show no significant differences in postoperative complications such as anastomotic fistulas, intraoperative bleeding, intestinal obstruction, urinary retention, urinary tract infections, urinary dysfunction, and abdominal infections between the robotic-assisted and laparoscopic groups. In conclusion, robot-assisted surgery is safe and feasible for the treatment of locally advanced mid-low rectal cancer after NCRT.

Positive CRM, distal resection margin, and the number of harvested lymph nodes are important indicators for evaluating the safety of tumour surgery. It is essential to ensure a negative resection margin and CRM, which is closely associated with lower rates of local recurrence and systemic recurrence. Based on two meta-analyses from randomised controlled trials, there were no differences in pathological outcomes between the robotic-assisted and laparoscopic groups, with the exception of the distal resection margin^53,54^. In colorectal surgery, there is a recommendation for the harvesting of at least 12 lymph nodes^55^. Except for the study by Lim *et al*.^35^, the number of harvested lymph nodes in the studies included in our meta-analysis exceeded 12, which may be associated with NCRT. In brief, our meta-analysis results show that the distal resection margin, positive CRM, and the number of harvested lymph nodes are all comparable in the two groups. As for long-term oncological outcomes, the most important indicators for evaluating the effectiveness of tumour treatment, our results indicate that there are no significant differences between the two groups. The results from the study by Zhang *et al*.^28^ show that the 3-year OS rates for the robotic-assisted and laparoscopic groups are 81.30 and 83.30%, respectively (*P*=0.640). The 3-year DFS rates for the robotic-assisted and laparoscopic groups are 77.70 and 75.90%, respectively (*P*=0.813). Additionally, results from studies by Piozzi *et al*.^31^, Chen and Liang^32^, Asoglu *et al*.^34^, and Lim *et al*.^35^ suggest that long-term oncological outcomes are comparable between the two groups. Therefore, the findings suggest that robot-assisted surgery is comparable to laparoscopic surgery in terms of oncological outcomes in patients with mid-low rectal cancer after NCRT.

Significant heterogeneity was observed in operative time, distal resection margin, and harvested number of lymph nodes. Sensitivity analysis and subgroup analysis results indicate that the study by Piozzi *et al*.^31^ maybe the source of heterogeneity in the total number of lymph nodes harvested. Other potential sources of heterogeneity may include study design and timing of surgery after NCRT. In the Western subgroup, there was no significant difference in operating time between the two groups, but there was significant heterogeneity. Reasons for this include possible bias due to the small number of included studies and possible reduction in operating time due to improved experience, implementation of standardized training, and use of precision technology. In addition, subgroup analyses suggest that the study design may have contributed to the high heterogeneity of distal resection margins. Sensitivity analyses also showed that the pooled results for these significant heterogeneity outcomes were stable, proving that the results were somewhat plausible.

Our study also has several limitations. Firstly, all 11 studies included were retrospective nonrandomised controlled trials, which may introduce potential biases. Secondly, relatively few trials and numbers of patients were included. Thirdly, most of the studies included in this research were from Asia and are not yet known to apply to other populations. Lastly, there are fewer studies investigating long-term oncological outcomes. Therefore, to confirm this in the future, more large and multicenter randomised controlled trials are needed.

## Conclusion

Compared to conventional laparoscopic surgery, robot-assisted surgery showed similar pathological outcomes, postoperative complications, and oncological outcomes. Robot-assisted surgery has the potential advantages, including resection of lower tumours with higher TME completion rates, lower open conversion rate, and shorter duration of hospitalisation, despite the longer operating time. Therefore, robotic-assisted surgery may be a safe, feasible, and effective option for patients with locally advanced mid-low rectal cancer patients after NCRT. However, future well-designed prospective studies are still a necessity to verify our findings.

## Ethical approval

Not applicable.

## Source of funding

Not applicable.

## Author contribution

X.-M.Z.: conceptualization, data curation, software, formal analysis, visualisation, methodology, and writing – original draft; X.B.: conceptualization, data curation, formal analysis, validation, methodology, and writing – review and editing; H.-Q.W.: visualisation, investigation, and writing – review and editing; D.D.: conceptualization, project administration, supervision, and writing – review and editing. All authors have read and agreed to the published version of the manuscript.

## Conflicts of interest disclosure

The authors declare no conflicts of interest.

## Research registration unique identifying number (UIN)

1. Name of the registry: PROSPERO database.
2. Unique identifying number or registration ID: CRD42024504498.
3. Hyperlink to your specific registration (must be publicly accessible and will be checked): https://www.crd.york.ac.uk/prospero/display_record.php?ID=CRD42024504498.

## Guarantor

Dong-Qiu Dai.

## Data availability statement

All data generated and analysed during this study are included in this article. The data supporting the findings of this study are available from the corresponding author upon reasonable request.

## Supplementary Material
