# Supplementary material for: Comparison of efficacy and safety between robotic-assisted versus laparoscopic surgery for locally advanced mid-low rectal cancer following neoadjuvant chemoradiotherapy: a systematic review and meta-analysis
Source: Int J Surg. 2024 Jun 24;111(1):1154–66. doi: 10.1097/JS9.0000000000001854 (PMC11745700; doi:10.1097/JS9.0000000000001854)
Supplement: Supplementary file 2 [file js9-111-1154-s002.doc]

**Identification of studies via databases and registers**

Records identified from Databases (n = 327):

PubMed (n = 105)

Embase (n = 20)

Web of science (n = 166)

Cochrane library (n = 36)

Records removed *before screening*:

Duplicate records removed (n = 131)

**Identification**

Records screened

(n = 196)

Records excluded after

reviewing the titles or abstracts

(n = 142)

(n = )

Reports sought for retrieval

(n = 54)

Reports not retrieved

(n = 0)

**Screening**

Reports assessed for eligibility

(n = 54)

Full-text articles excluded, with reasons as follows (n = 43)

No neoadjuvant therapy (n = 39)

Non-advanced mid-low rectal cancer (n = 1)

Non-comparative study (n = 1)

Date repetition (n = 2)

Studies included in review

(n = 11)

**Included**

*Consider, if feasible to do so, reporting the number of records identified from each database or register searched (rather than the total number across all databases/registers).

**If automation tools were used, indicate how many records were excluded by a human and how many were excluded by automation tools.

*From:*  Page MJ, McKenzie JE, Bossuyt PM, Boutron I, Hoffmann TC, Mulrow CD, et al. The PRISMA 2020 statement: an updated guideline for reporting systematic reviews. BMJ 2021;372:n71. doi: 10.1136/bmj.n71

For more information, visit: <http://www.prisma-statement.org/>
