# Supplementary material for: Comparison of efficacy and safety between robotic-assisted versus laparoscopic surgery for locally advanced mid-low rectal cancer following neoadjuvant chemoradiotherapy: a systematic review and meta-analysis
Source: Int J Surg. 2024 Jun 24;111(1):1154–66. doi: 10.1097/JS9.0000000000001854 (PMC11745700; doi:10.1097/JS9.0000000000001854)
Supplement: Supplementary file 4 [file js9-111-1154-s004.pdf]

Comparison of efficacy and safety between robotic-assisted versus laparoscopic surgery for locally advanced mid-low rectal cancer following neoadjuvant chemoradiotherapy: A systematic review and meta-analysis

Xin-Mao Zhu

Table S1. Search strategy.

**PubMed: 105**	
#1	(Neoadjuvant Therapy[MeSH Terms]) OR (Neoadjuvant Therapy[Title/Abstract] OR Neoadjuvant Therapies[Title/Abstract] OR Therapy, Neoadjuvant[Title/Abstract] OR Neoadjuvant Treatment[Title/Abstract] OR Neoadjuvant Treatments[Title/Abstract] OR Treatment, Neoadjuvant[Title/Abstract] OR Neoadjuvant Chemoradiotherapy[Title/Abstract] OR Chemoradiotherapy, Neoadjuvant[Title/Abstract] OR Neoadjuvant Chemoradiotherapies[Title/Abstract] OR Neoadjuvant Chemoradiation Therapy[Title/Abstract] OR Chemoradiation Therapy, Neoadjuvant[Title/Abstract] OR Neoadjuvant Chemoradiation Therapies[Title/Abstract] OR Therapy, Neoadjuvant Chemoradiation[Title/Abstract] OR Neoadjuvant Chemoradiation Treatment[Title/Abstract] OR Chemoradiation Treatment, Neoadjuvant[Title/Abstract] OR Neoadjuvant Chemoradiation Treatments[Title/Abstract] OR Treatment, Neoadjuvant Chemoradiation[Title/Abstract] OR Neoadjuvant Chemoradiation[Title/Abstract] OR Chemoradiation, Neoadjuvant[Title/Abstract] OR Neoadjuvant Chemoradiations[Title/Abstract] OR Neoadjuvant Radiotherapy[Title/Abstract] OR Neoadjuvant Radiotherapies[Title/Abstract] OR Radiotherapy, Neoadjuvant[Title/Abstract] OR Neoadjuvant Radiation Treatment[Title/Abstract] OR Neoadjuvant Radiation Treatments[Title/Abstract] OR Radiation Treatment, Neoadjuvant[Title/Abstract] OR Treatment, Neoadjuvant Radiation[Title/Abstract] OR Neoadjuvant Radiation Therapy[Title/Abstract] OR Neoadjuvant Radiation Therapies[Title/Abstract] OR Radiation Therapy, Neoadjuvant[Title/Abstract] OR Therapy, Neoadjuvant Radiation[Title/Abstract] OR Neoadjuvant Radiation[Title/Abstract] OR Neoadjuvant Radiations[Title/Abstract] OR Radiation, Neoadjuvant[Title/Abstract] OR Neoadjuvant Chemotherapy[Title/Abstract] OR Chemotherapy, Neoadjuvant[Title/Abstract] OR Neoadjuvant Chemotherapies[Title/Abstract] OR Neoadjuvant Chemotherapy Treatment[Title/Abstract] OR Chemotherapy Treatment, Neoadjuvant[Title/Abstract] OR Neoadjuvant Chemotherapy Treatments[Title/Abstract] OR Treatment, Neoadjuvant Chemotherapy[Title/Abstract] OR Neoadjuvant Systemic Therapy[Title/Abstract] OR Neoadjuvant Systemic Therapies[Title/Abstract] OR Systemic Therapy, Neoadjuvant[Title/Abstract] OR Therapy, Neoadjuvant

	Systemic[Title/Abstract] OR Neoadjuvant Systemic Treatment[Title/Abstract] OR Neoadjuvant Systemic Treatments[Title/Abstract] OR Systemic Treatment, Neoadjuvant[Title/Abstract] OR Treatment, Neoadjuvant Systemic[Title/Abstract] OR Preoperative therapy[Title/Abstract] OR Preoperative Chemoradiotherapy[Title/Abstract] OR Preoperative Chemotherapy[Title/Abstract] OR preoperative radiotherapy[Title/Abstract] OR Preoperative therapies[Title/Abstract] OR Preoperative chemoradiotherapies[Title/Abstract] OR Preoperative Chemotherapies[Title/Abstract] OR preoperative radiotherapies[Title/Abstract])
#2	(Rectal Neoplasms[MeSH Terms]) OR (Rectal Neoplasms[Title/Abstract] OR Neoplasm, Rectal[Title/Abstract] OR Rectal Neoplasm[Title/Abstract] OR Rectum Neoplasms[Title/Abstract] OR Neoplasm, Rectum[Title/Abstract] OR Rectum Neoplasm[Title/Abstract] OR Rectal Tumors[Title/Abstract] OR Rectal Tumor[Title/Abstract] OR Tumor, Rectal[Title/Abstract] OR Neoplasms, Rectal[Title/Abstract] OR Cancer of Rectum[Title/Abstract] OR Rectum Cancers[Title/Abstract] OR Rectal Cancer[Title/Abstract] OR Cancer, Rectal[Title/Abstract] OR Rectal Cancers[Title/Abstract] OR Rectum Cancer[Title/Abstract] OR Cancer, Rectum[Title/Abstract] OR Cancer of the Rectum[Title/Abstract])
#3	(Robotic Surgical Procedures[MeSH Terms]) OR (Robotic Surgical Procedures[Title/Abstract] OR Procedure, Robotic Surgical[Title/Abstract] OR Procedures, Robotic Surgical[Title/Abstract] OR Robotic Surgical Procedure[Title/Abstract] OR Surgical Procedure, Robotic[Title/Abstract] OR Robot Surgery[Title/Abstract] OR Robot Surgeries[Title/Abstract] OR Surgery, Robot[Title/Abstract] OR Robot-Assisted Surgery[Title/Abstract] OR Robot Assisted Surgery[Title/Abstract] OR Robot-Assisted Surgeries[Title/Abstract] OR Surgery, Robot-Assisted[Title/Abstract] OR Robot-Enhanced Procedures[Title/Abstract] OR Procedure, Robot-Enhanced[Title/Abstract] OR Robot Enhanced Procedures[Title/Abstract] OR Robot-Enhanced Procedure[Title/Abstract] OR Surgical Procedures, Robotic[Title/Abstract] OR Robotic-Assisted Surgery[Title/Abstract] OR Robotic Assisted Surgery[Title/Abstract] OR Robotic-Assisted Surgeries[Title/Abstract] OR Surgery, Robotic-Assisted[Title/Abstract] OR Robot-Enhanced Surgery[Title/Abstract] OR Robot Enhanced Surgery[Title/Abstract] OR Robot-Enhanced Surgeries[Title/Abstract] OR Surgery, Robot-Enhanced[Title/Abstract])
#4	Laparoscopy[MeSH Terms] OR Laparoscopy[Title/Abstract] OR Laparoscopies[Title/Abstract] OR Celioscopy[Title/Abstract] OR Celioscopies[Title/Abstract] OR Peritoneoscopy[Title/Abstract] OR Peritoneoscopies[Title/Abstract] OR Surgical Procedures, Laparoscopic[Title/Abstract] OR Laparoscopic Surgical

	Procedure[Title/Abstract] OR Procedure, Laparoscopic Surgical[Title/Abstract] OR Procedures, Laparoscopic Surgical[Title/Abstract] OR Surgery, Laparoscopic[Title/Abstract] OR Laparoscopic Surgical Procedures[Title/Abstract] OR Laparoscopic Surgery[Title/Abstract] OR Laparoscopic Surgeries[Title/Abstract] OR Surgeries, Laparoscopic[Title/Abstract] OR Laparoscopic Assisted Surgery[Title/Abstract] OR Laparoscopic Assisted Surgeries[Title/Abstract] OR Surgeries, Laparoscopic Assisted[Title/Abstract] OR Surgery, Laparoscopic Assisted[Title/Abstract] OR Surgical Procedure, Laparoscopic[Title/Abstract]
#5	#1 AND #2 AND #3 AND #4
**Embase: 20**	
#1	'neoadjuvant therapy':ti,ab,kw OR 'neoadjuvant therapies':ti,ab,kw OR 'therapy, neoadjuvant':ti,ab,kw OR 'neoadjuvant treatment':ti,ab,kw OR 'neoadjuvant treatments':ti,ab,kw OR 'treatment, neoadjuvant':ti,ab,kw OR 'neoadjuvant chemoradiotherapy':ti,ab,kw OR 'chemoradiotherapy, neoadjuvant':ti,ab,kw OR 'neoadjuvant chemoradiotherapies':ti,ab,kw OR 'neoadjuvant chemoradiation therapy':ti,ab,kw OR 'chemoradiation therapy, neoadjuvant':ti,ab,kw OR 'neoadjuvant chemoradiation therapies':ti,ab,kw OR 'therapy, neoadjuvant chemoradiation':ti,ab,kw OR 'neoadjuvant chemoradiation treatment':ti,ab,kw OR 'chemoradiation treatment, neoadjuvant':ti,ab,kw OR 'neoadjuvant chemoradiation treatments':ti,ab,kw OR 'treatment, neoadjuvant chemoradiation':ti,ab,kw OR 'neoadjuvant chemoradiation':ti,ab,kw OR 'chemoradiation, neoadjuvant':ti,ab,kw OR 'neoadjuvant chemoradiations':ti,ab,kw OR 'neoadjuvant radiotherapy':ti,ab,kw OR 'neoadjuvant radiotherapies':ti,ab,kw OR 'radiotherapy, neoadjuvant':ti,ab,kw OR 'neoadjuvant radiation treatment':ti,ab,kw OR 'neoadjuvant radiation treatments':ti,ab,kw OR 'radiation treatment, neoadjuvant':ti,ab,kw OR 'treatment, neoadjuvant radiation':ti,ab,kw OR 'neoadjuvant radiation therapy':ti,ab,kw OR 'neoadjuvant radiation therapies':ti,ab,kw OR 'radiation therapy, neoadjuvant':ti,ab,kw OR 'therapy, neoadjuvant radiation':ti,ab,kw OR 'neoadjuvant radiation':ti,ab,kw OR 'neoadjuvant radiations':ti,ab,kw OR 'radiation, neoadjuvant':ti,ab,kw OR 'neoadjuvant chemotherapy':ti,ab,kw OR 'chemotherapy, neoadjuvant':ti,ab,kw OR 'neoadjuvant chemotherapies':ti,ab,kw OR 'neoadjuvant chemotherapy treatment':ti,ab,kw OR 'chemotherapy treatment, neoadjuvant':ti,ab,kw OR 'neoadjuvant chemotherapy treatments':ti,ab,kw OR 'treatment, neoadjuvant chemotherapy':ti,ab,kw OR 'neoadjuvant systemic therapy':ti,ab,kw OR 'neoadjuvant systemic therapies':ti,ab,kw OR 'systemic therapy, neoadjuvant':ti,ab,kw OR 'therapy, neoadjuvant systemic':ti,ab,kw OR 'neoadjuvant systemic treatment':ti,ab,kw OR 'neoadjuvant systemic treatments':ti,ab,kw OR 'systemic treatment, neoadjuvant':ti,ab,kw OR 'treatment, neoadjuvant systemic':ti,ab,kw OR 'preoperative therapy':ti,ab,kw OR 'preoperative

	chemoradiotherapy':ti,ab,kw OR 'preoperative chemotherapy':ti,ab,kw OR 'preoperative radiotherapy':ti,ab,kw OR 'preoperative therapies':ti,ab,kw OR 'preoperative chemoradiotherapies':ti,ab,kw OR 'preoperative chemotherapies':ti,ab,kw OR 'preoperative radiotherapies':ti,ab,kw
#2	'rectal neoplasms':ti,ab,kw OR 'neoplasm, rectal':ti,ab,kw OR 'rectal neoplasm':ti,ab,kw OR 'rectum neoplasms':ti,ab,kw OR 'neoplasm, rectum':ti,ab,kw OR 'rectum neoplasm':ti,ab,kw OR 'rectal tumors':ti,ab,kw OR 'rectal tumor':ti,ab,kw OR 'tumor, rectal':ti,ab,kw OR 'neoplasms, rectal':ti,ab,kw OR 'cancer of rectum':ti,ab,kw OR 'rectum cancers':ti,ab,kw OR 'rectal cancer':ti,ab,kw OR 'cancer, rectal':ti,ab,kw OR 'rectal cancers':ti,ab,kw OR 'cancer, rectum':ti,ab,kw OR 'rectum cancer':ti,ab,kw
#3	'robotic surgical procedures':ti,ab,kw OR 'procedure, robotic surgical':ti,ab,kw OR 'procedures, robotic surgical':ti,ab,kw OR 'robotic surgical procedure':ti,ab,kw OR 'surgical procedure, robotic':ti,ab,kw OR 'robot surgery':ti,ab,kw OR 'robot surgeries':ti,ab,kw OR 'surgery, robot':ti,ab,kw OR 'robot-assisted surgery':ti,ab,kw OR 'robot assisted surgery':ti,ab,kw OR 'robot-assisted surgeries':ti,ab,kw OR 'surgery, robot-assisted':ti,ab,kw OR 'robot-enhanced procedures':ti,ab,kw OR 'procedure, robot-enhanced':ti,ab,kw OR 'robot enhanced procedures':ti,ab,kw OR 'robot-enhanced procedure':ti,ab,kw OR 'surgical procedures, robotic':ti,ab,kw OR 'robotic-assisted surgery':ti,ab,kw OR 'robotic assisted surgery':ti,ab,kw OR 'robotic-assisted surgeries':ti,ab,kw OR 'surgery, robotic-assisted':ti,ab,kw OR 'robot-enhanced surgery':ti,ab,kw OR 'robot enhanced surgery':ti,ab,kw OR 'robot-enhanced surgeries':ti,ab,kw OR 'surgery, robot-enhanced':ti,ab,kw
#4	laparoscopy:ti,ab,kw OR laparoscopies:ti,ab,kw OR celioscopy:ti,ab,kw OR celioscopies:ti,ab,kw OR peritoneoscopy:ti,ab,kw OR peritoneoscopies:ti,ab,kw OR 'surgical procedures, laparoscopic':ti,ab,kw OR 'laparoscopic surgical procedure':ti,ab,kw OR 'procedure, laparoscopic surgical':ti,ab,kw OR 'procedures, laparoscopic surgical':ti,ab,kw OR 'surgery, laparoscopic':ti,ab,kw OR 'laparoscopic surgical procedures':ti,ab,kw OR 'laparoscopic surgery':ti,ab,kw OR 'laparoscopic surgeries':ti,ab,kw OR 'surgeries, laparoscopic':ti,ab,kw OR 'laparoscopic assisted surgery':ti,ab,kw OR 'laparoscopic assisted surgeries':ti,ab,kw OR 'surgeries, laparoscopic assisted':ti,ab,kw OR 'surgery, laparoscopic assisted':ti,ab,kw OR 'surgical procedure, laparoscopic':ti,ab,kw
#5	#1 AND #2 AND #3 AND #4

Web of science: 166	
#1	(AB=(Neoadjuvant Therapy OR Neoadjuvant Therapies OR Therapy, Neoadjuvant OR Neoadjuvant Treatment OR Neoadjuvant Treatments OR Treatment, Neoadjuvant OR Neoadjuvant Chemoradiotherapy OR Chemoradiotherapy, Neoadjuvant OR Neoadjuvant Chemoradiotherapies OR Neoadjuvant Chemoradiation Therapy OR Chemoradiation Therapy, Neoadjuvant OR Neoadjuvant Chemoradiation Therapies OR Therapy, Neoadjuvant Chemoradiation OR Neoadjuvant Chemoradiation Treatment OR Chemoradiation Treatment, Neoadjuvant OR Neoadjuvant Chemoradiation Treatments OR Treatment, Neoadjuvant Chemoradiation OR Neoadjuvant Chemoradiation OR Chemoradiation, Neoadjuvant OR Neoadjuvant Chemoradiations OR Neoadjuvant Radiotherapy OR Neoadjuvant Radiotherapies OR Radiotherapy, Neoadjuvant OR Neoadjuvant Radiation Treatment OR Neoadjuvant Radiation Treatments OR Radiation Treatment, Neoadjuvant OR Treatment, Neoadjuvant Radiation OR Neoadjuvant Radiation Therapy OR Neoadjuvant Radiation Therapies OR Radiation Therapy, Neoadjuvant OR Therapy, Neoadjuvant Radiation OR Neoadjuvant Radiation OR Neoadjuvant Radiations OR Radiation, Neoadjuvant OR Neoadjuvant Chemotherapy OR Chemotherapy, Neoadjuvant OR Neoadjuvant Chemotherapies OR Neoadjuvant Chemotherapy Treatment OR Chemotherapy Treatment, Neoadjuvant OR Neoadjuvant Chemotherapy Treatments OR Treatment, Neoadjuvant Chemotherapy OR Neoadjuvant Systemic Therapy OR Neoadjuvant Systemic Therapies OR Systemic Therapy, Neoadjuvant OR Therapy, Neoadjuvant Systemic OR Neoadjuvant Systemic Treatment OR Neoadjuvant Systemic Treatments OR Systemic Treatment, Neoadjuvant OR Treatment, Neoadjuvant Systemic OR Preoperative therapy OR Preoperative Chemoradiotherapy OR Preoperative Chemotherapy OR preoperative radiotherapy OR Preoperative therapies OR Preoperative chemoradiotherapies OR Preoperative Chemotherapies OR preoperative radiotherapies) OR TS=Neoadjuvant Therapy)
#2	(AB=(Rectal Neoplasms OR Neoplasm, Rectal OR Rectal Neoplasm OR Rectum Neoplasms OR Neoplasm, Rectum OR Rectum Neoplasm OR Rectal Tumors OR Rectal Tumor OR Tumor, Rectal OR Neoplasms, Rectal OR Cancer of Rectum OR Rectum Cancers OR Rectal Cancer OR Cancer, Rectal OR Rectal Cancers OR Rectum Cancer OR Cancer, Rectum OR Cancer of the Rectum) OR TS=Rectal Neoplasms)
#3	(AB=(Robotic Surgical Procedures OR Procedure, Robotic Surgical OR Procedures, Robotic Surgical OR Robotic Surgical Procedure OR Surgical Procedure, Robotic OR Robot Surgery OR Robot Surgeries OR Surgery, Robot OR Robot-Assisted Surgery OR Robot Assisted Surgery OR Robot-Assisted Surgeries OR Surgery, Robot-Assisted OR Robot-Enhanced Procedures OR Procedure, Robot-Enhanced OR Robot Enhanced Procedures OR Robot-Enhanced Procedure OR Surgical Procedures, Robotic OR Robotic-Assisted Surgery OR Robotic Assisted Surgery OR

	Robotic-Assisted Surgeries OR Surgery, Robotic-Assisted OR Robot-Enhanced Surgery OR Robot Enhanced Surgery OR Robot-Enhanced Surgeries OR Surgery, Robot-Enhanced) OR TS=Robotic Surgical Procedures)
#4	(AB=(Laparoscopy OR Laparoscopies OR Celioscopy OR Celioscopies OR Peritoneoscopy OR Peritoneoscopies OR Surgical Procedures, Laparoscopic OR Laparoscopic Surgical Procedure OR Procedure, Laparoscopic Surgical OR Procedures, Laparoscopic Surgical OR Surgery, Laparoscopic OR Laparoscopic Surgical Procedures OR Laparoscopic Surgery OR Laparoscopic Surgeries OR Surgeries, Laparoscopic OR Laparoscopic Assisted Surgery OR Laparoscopic Assisted Surgeries OR Surgeries, Laparoscopic Assisted OR Surgery, Laparoscopic Assisted OR Surgical Procedure, Laparoscopic) OR TS=Laparoscopy)
#5	#1 AND #2 AND #3 AND #4
**Cochrane library: 36**	
#1	(Neoadjuvant Therapy OR Neoadjuvant Therapies OR Therapy, Neoadjuvant OR Neoadjuvant Treatment OR Neoadjuvant Treatments OR Treatment, Neoadjuvant OR Neoadjuvant Chemoradiotherapy OR Chemoradiotherapy, Neoadjuvant OR Neoadjuvant Chemoradiotherapies OR Neoadjuvant Chemoradiation Therapy OR Chemoradiation Therapy, Neoadjuvant OR Neoadjuvant Chemoradiation Therapies OR Therapy, Neoadjuvant Chemoradiation OR Neoadjuvant Chemoradiation Treatment OR Chemoradiation Treatment, Neoadjuvant OR Neoadjuvant Chemoradiation Treatments OR Treatment, Neoadjuvant Chemoradiation OR Neoadjuvant Chemoradiation OR Chemoradiation, Neoadjuvant OR Neoadjuvant Chemoradiations OR Neoadjuvant Radiotherapy OR Neoadjuvant Radiotherapies OR Radiotherapy, Neoadjuvant OR Neoadjuvant Radiation Treatment OR Neoadjuvant Radiation Treatments OR Radiation Treatment, Neoadjuvant OR Treatment, Neoadjuvant Radiation OR Neoadjuvant Radiation Therapy OR Neoadjuvant Radiation Therapies OR Radiation Therapy, Neoadjuvant OR Therapy, Neoadjuvant Radiation OR Neoadjuvant Radiation OR Neoadjuvant Radiations OR Radiation, Neoadjuvant OR Neoadjuvant Chemotherapy OR Chemotherapy, Neoadjuvant OR Neoadjuvant Chemotherapies OR Neoadjuvant Chemotherapy Treatment OR Chemotherapy Treatment, Neoadjuvant OR Neoadjuvant Chemotherapy Treatments OR Treatment, Neoadjuvant Chemotherapy OR Neoadjuvant Systemic Therapy OR Neoadjuvant Systemic Therapies OR Systemic Therapy, Neoadjuvant OR Therapy, Neoadjuvant Systemic OR Neoadjuvant Systemic Treatment OR Neoadjuvant Systemic Treatments OR Systemic Treatment, Neoadjuvant OR Treatment, Neoadjuvant Systemic OR Preoperative therapy OR Preoperative Chemoradiotherapy OR Preoperative Chemotherapy OR preoperative radiotherapy OR Preoperative therapies OR Preoperative chemoradiotherapies OR Preoperative

	Chemotherapies OR preoperative radiotherapies):ti,ab,kw
#2	(Rectal Neoplasms OR Neoplasm, Rectal OR Rectal Neoplasm OR Rectum Neoplasms OR Neoplasm, Rectum OR Rectum Neoplasm OR Rectal Tumors OR Rectal Tumor OR Tumor, Rectal OR Neoplasms, Rectal OR Cancer of Rectum OR Rectum Cancers OR Rectal Cancer OR Cancer, Rectal OR Rectal Cancers OR Rectum Cancer OR Cancer, Rectum OR Cancer of the Rectum):ti,ab,kw
#3	(Robotic Surgical Procedures OR Procedure, Robotic Surgical OR Procedures, Robotic Surgical OR Robotic Surgical Procedure OR Surgical Procedure, Robotic OR Robot Surgery OR Robot Surgeries OR Surgery, Robot OR Robot-Assisted Surgery OR Robot Assisted Surgery OR Robot-Assisted Surgeries OR Surgery, Robot-Assisted OR Robot-Enhanced Procedures OR Procedure, Robot-Enhanced OR Robot Enhanced Procedures OR Robot-Enhanced Procedure OR Surgical Procedures, Robotic OR Robotic-Assisted Surgery OR Robotic Assisted Surgery OR Robotic-Assisted Surgeries OR Surgery, Robotic-Assisted OR Robot-Enhanced Surgery OR Robot Enhanced Surgery OR Robot-Enhanced Surgeries OR Surgery, Robot-Enhanced):ti,ab,kw
#4	(Laparoscopy OR Laparoscopies OR Celioscopy OR Celioscopies OR Peritoneoscopy OR Peritoneoscopies OR Surgical Procedures, Laparoscopic OR Laparoscopic Surgical Procedure OR Procedure, Laparoscopic Surgical OR Procedures, Laparoscopic Surgical OR Surgery, Laparoscopic OR Laparoscopic Surgical Procedures OR Laparoscopic Surgery OR Laparoscopic Surgeries OR Surgeries, Laparoscopic OR Laparoscopic Assisted Surgery OR Laparoscopic Assisted Surgeries OR Surgeries, Laparoscopic Assisted OR Surgery, Laparoscopic Assisted OR Surgical Procedure, Laparoscopic):ti,ab,kw
#5	#1 AND #2 AND #3 AND #4

Table S2. Quality assessments of included studies by Newcastle-Ottawa Scale.

References	Selection				Comparability	Exposure			Total score	Assessment
	Definition of cases	Representativeness of cases	Selection of Controls	Definition of Controls		Ascertainment of exposure	Same method of ascertainment	Non-response rate		
Zhang L et al. 2023 [28]	1	1	1	1	2	1	1	_	8	High
Yamanashi T et al. 2023 [19]	1	1	1	1	2	1	1	1	9	High
Lim S et al. 2023 [29]	1	1	_	1	2	1	1	_	7	High
Ishizaki T et al. 2023 [30]	1	1	_	1	2	1	1	_	7	High
Piozzi GN et al. 2022 [31]	1	1	1	1	2	1	1	1	9	High
Chen TC et al. 2022 [32]	1	1	_	1	2	1	1	1	8	High
Angehrn FV et al. 2022 [33]	1	1	1	1	2	1	1	1	9	High
Asoglu O et al. 2020 [34]	1	1	1	1	2	1	1	1	9	High
Lim DR et al. 2017 [35]	1	1	1	1	2	1	1	_	8	High
Huang YM et al. 2017 [36]	1	1	_	1	2	1	1	_	7	High
Kim YS et al. 2016 [37]	1	1	1	1	2	1	1	1	9	High

_, with a Newcastle-Ottawa Scale score of 0.
