# Supplementary material for: Comparison of efficacy and safety between robotic-assisted versus laparoscopic surgery for locally advanced mid-low rectal cancer following neoadjuvant chemoradiotherapy: a systematic review and meta-analysis
Source: Int J Surg. 2024 Jun 24;111(1):1154–66. doi: 10.1097/JS9.0000000000001854 (PMC11745700; doi:10.1097/JS9.0000000000001854)
Supplement: Supplementary file 5 [file js9-111-1154-s005.pdf]

Comparison of efficacy and safety between robotic-assisted versus laparoscopic surgery for locally advanced mid-low rectal cancer following neoadjuvant chemoradiotherapy: A systematic review and meta-analysis

Xin-Mao Zhu

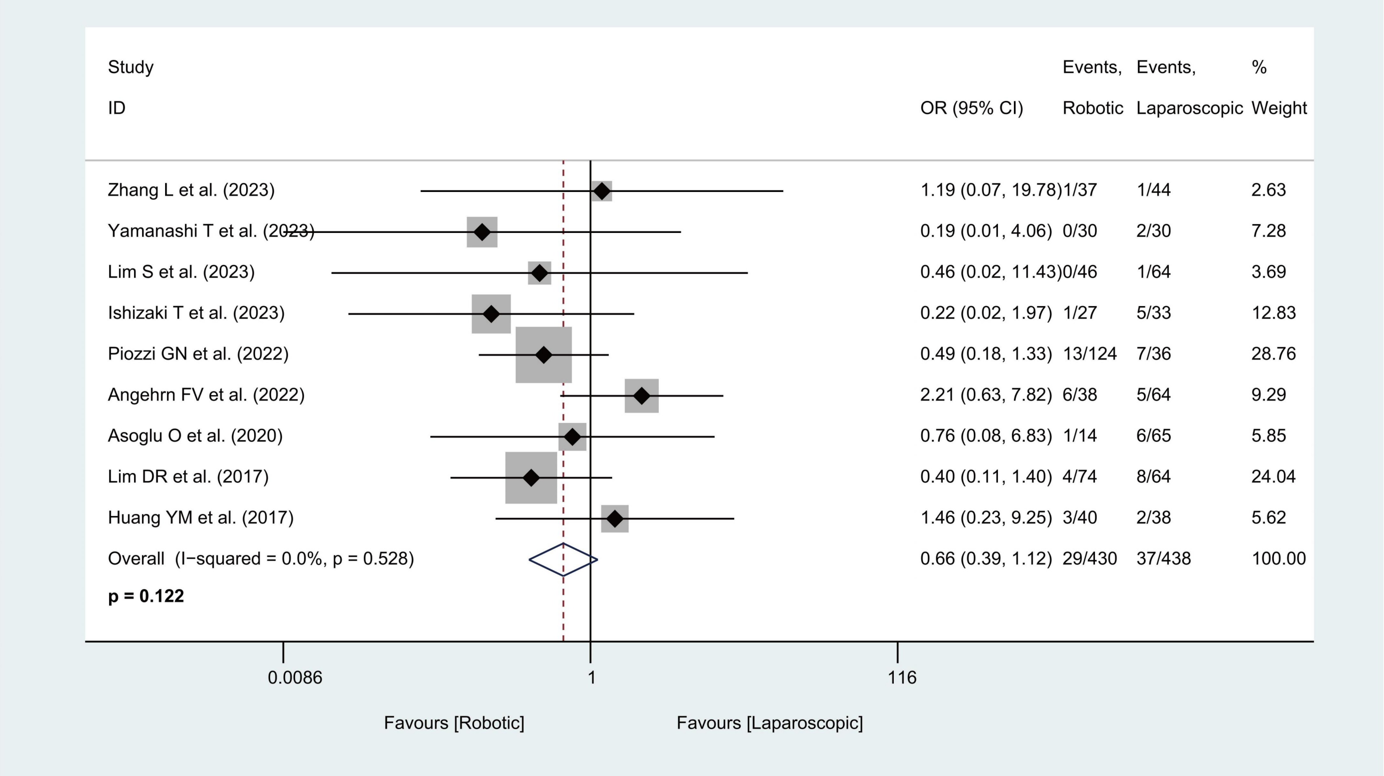

Figure S1. Forest plot of anastomosis leakage in the robotic and laparoscopic groups. Abbreviation: OR, odds ratio; CI, confidence interval.

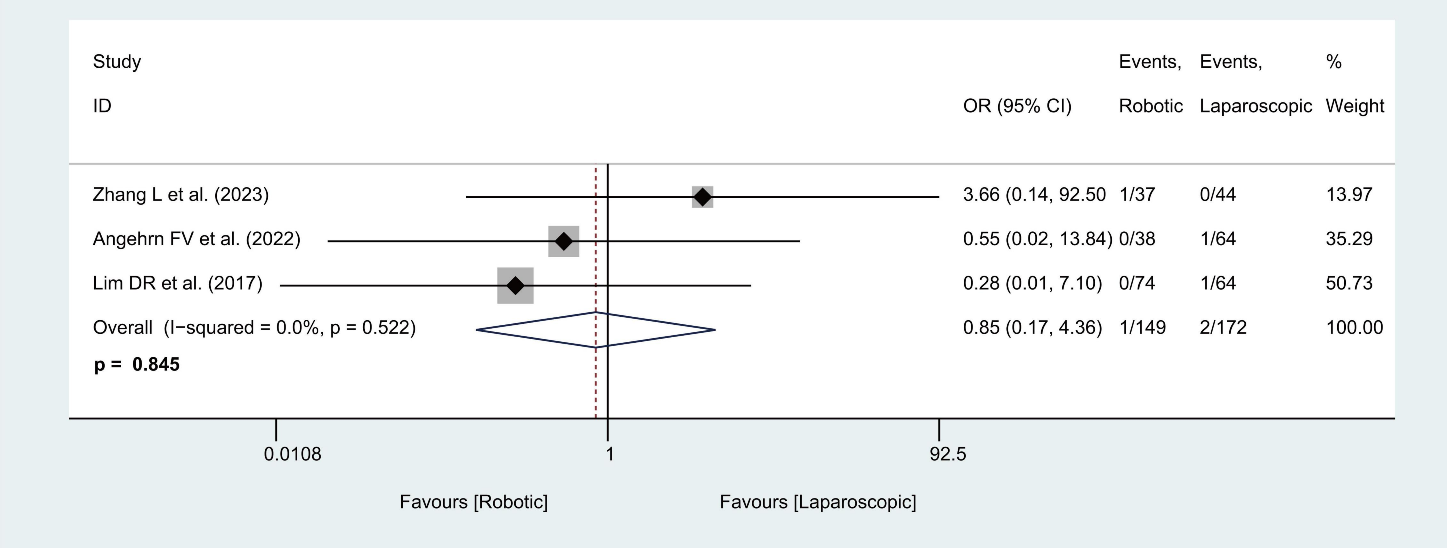

Figure S2. Forest plot of postoperative bleeding in the robotic and laparoscopic groups. Abbreviation: OR, odds ratio; CI, confidence interval.

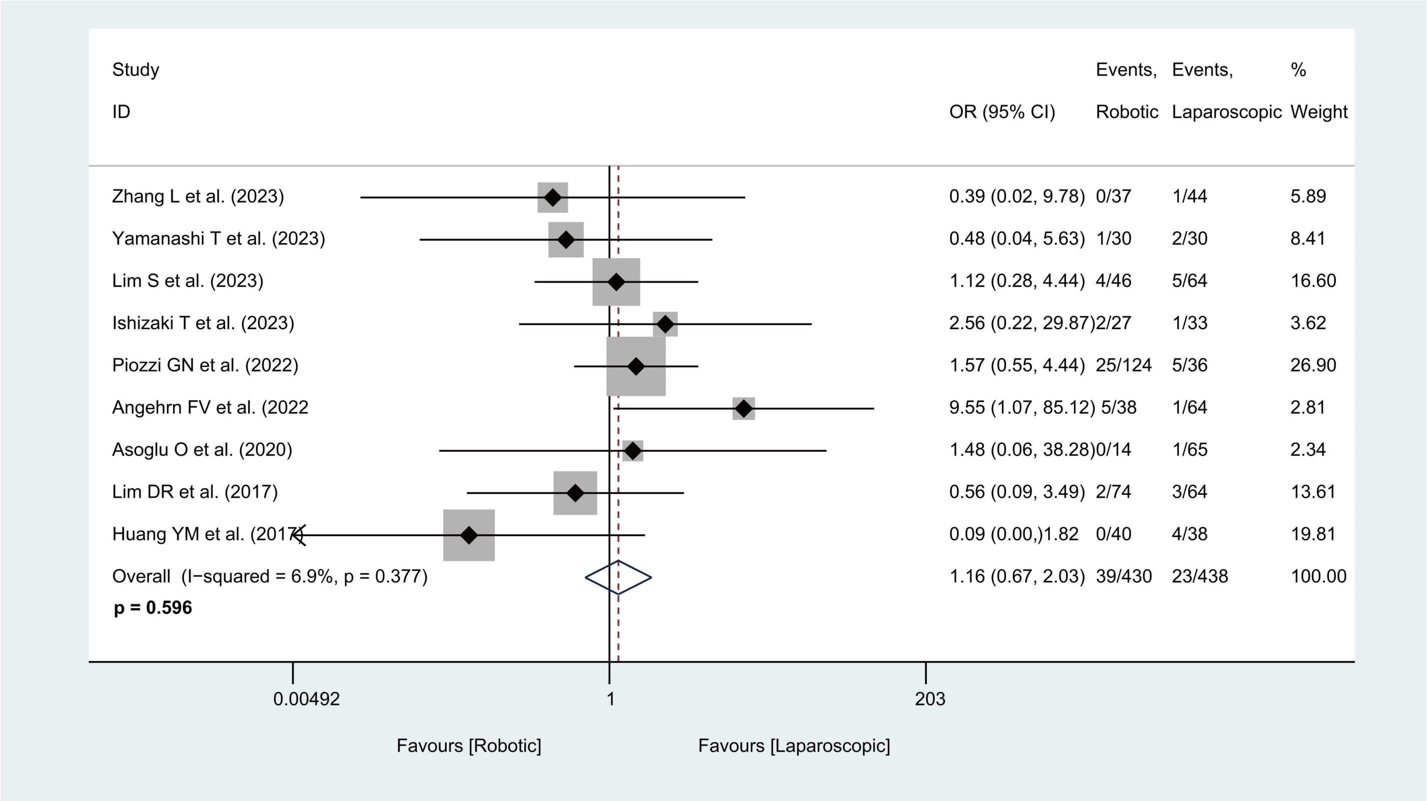

Figure S3. Forest plot of postoperative ileus in the robotic and laparoscopic groups. Abbreviation: OR, odds ratio; CI, confidence interval.

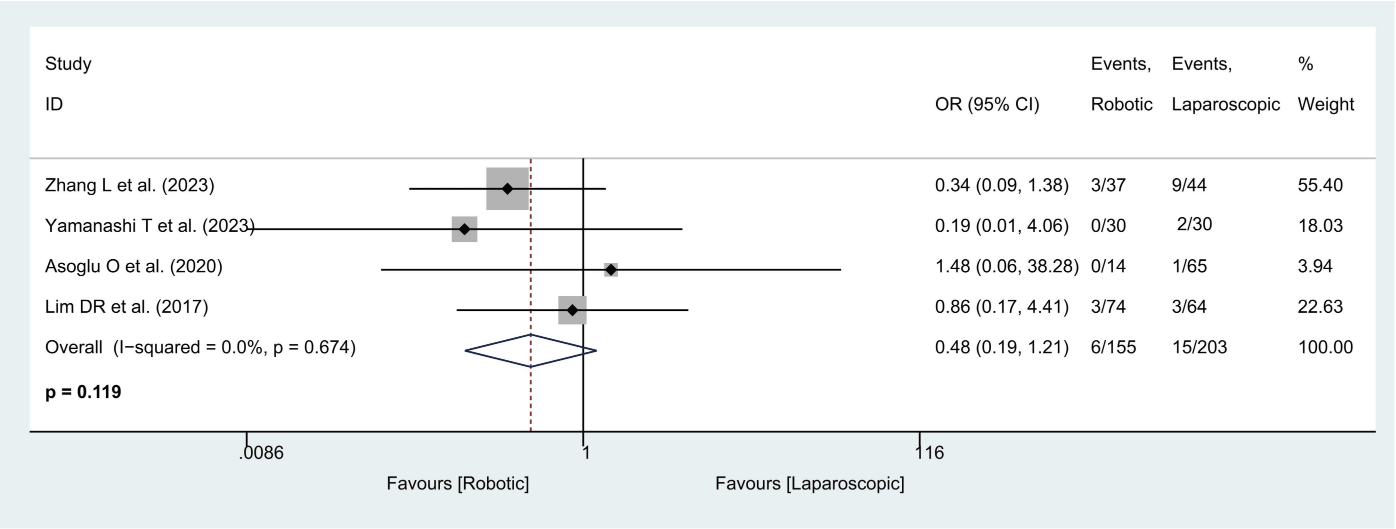

Figure S4. Forest plot of urine retention in the robotic and laparoscopic groups. Abbreviation: OR, odds ratio; CI, confidence interval.

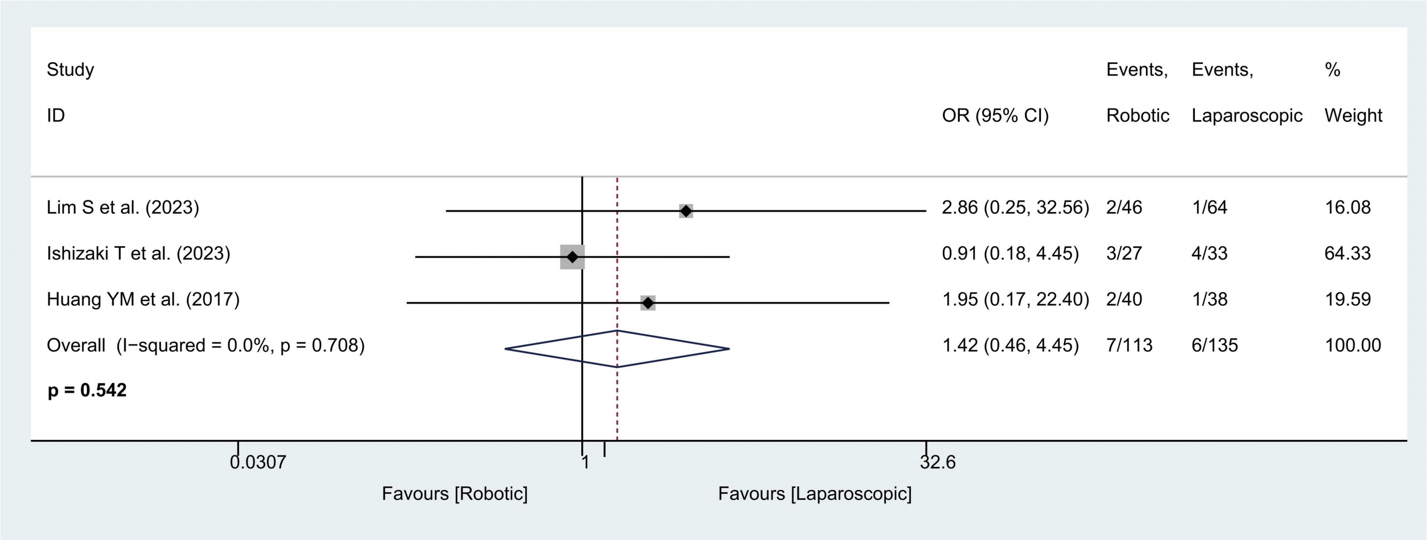

Figure S5. Forest plot of urinary dysfunction in the robotic and laparoscopic groups. Abbreviation: OR, odds ratio; CI, confidence interval.

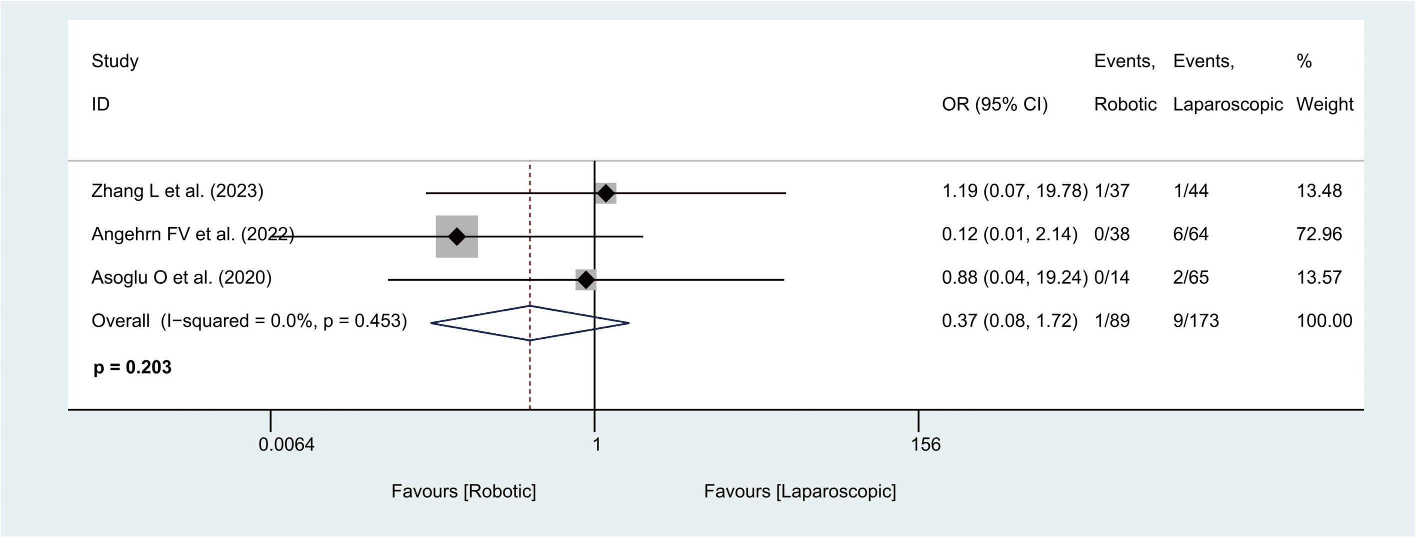

Figure S6. Forest plot of urinary infection in the robotic and laparoscopic groups. Abbreviation: OR, odds ratio; CI, confidence interval.

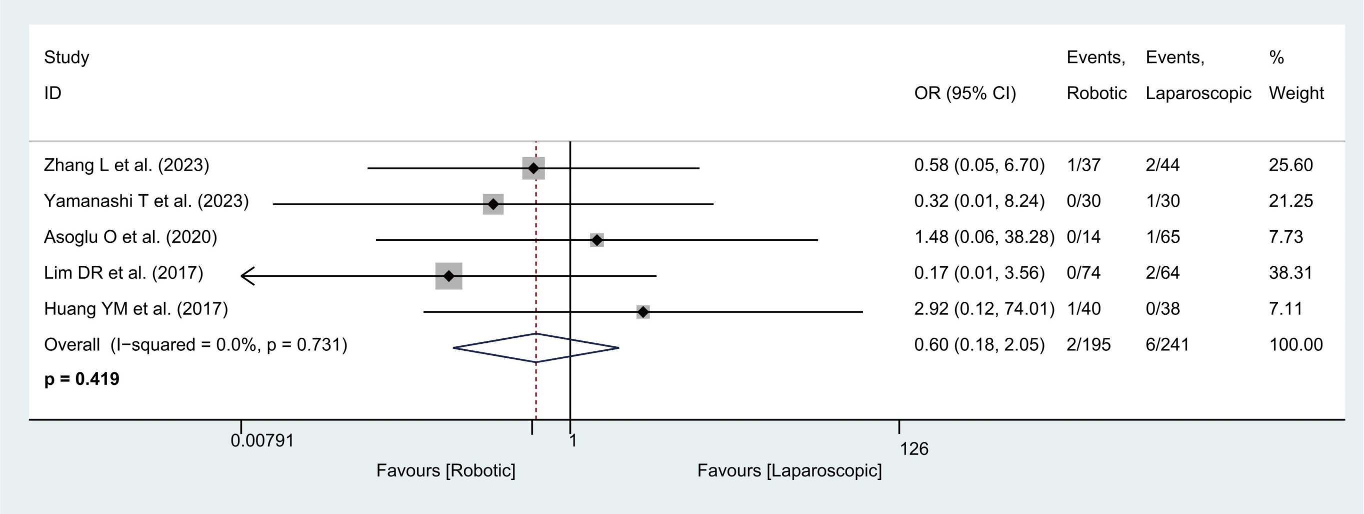

Figure S7. Forest plot of intra-abdominal infection in the robotic and laparoscopic groups.

Abbreviation: OR, odds ratio; CI, confidence interval.

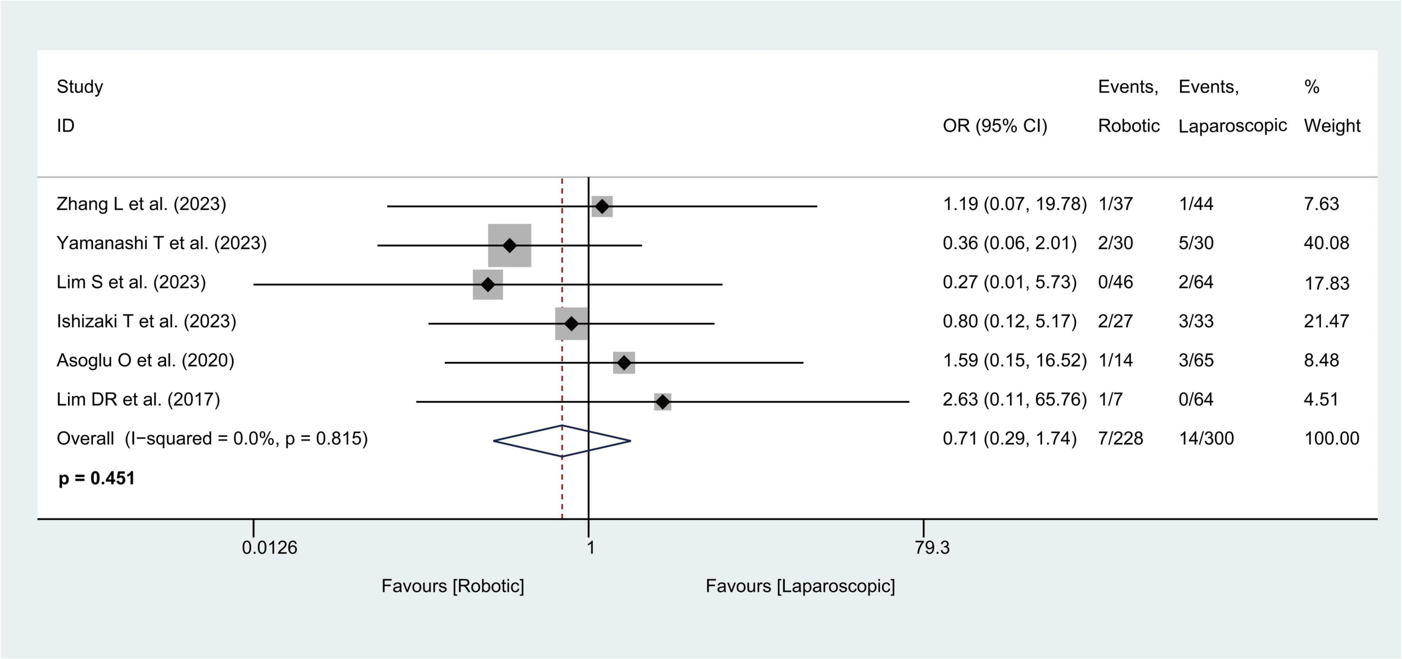

Figure S8. Forest plot of surgical site infection in the robotic and laparoscopic groups. Abbreviation: OR, odds ratio; CI, confidence interval.

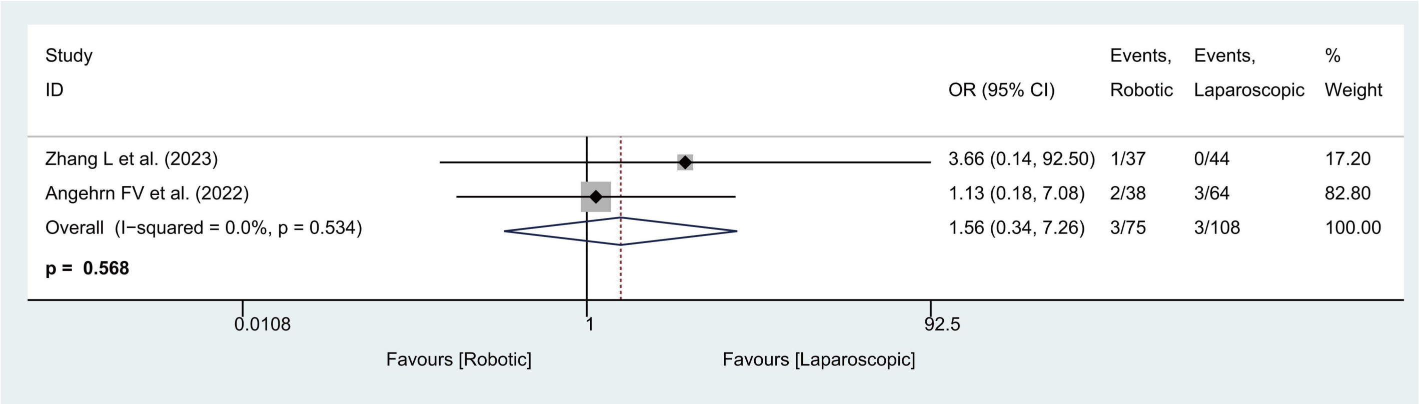

Figure S9. Forest plot of pulmonary complication in the robotic and laparoscopic groups. Abbreviation: OR, odds ratio; CI, confidence interval.

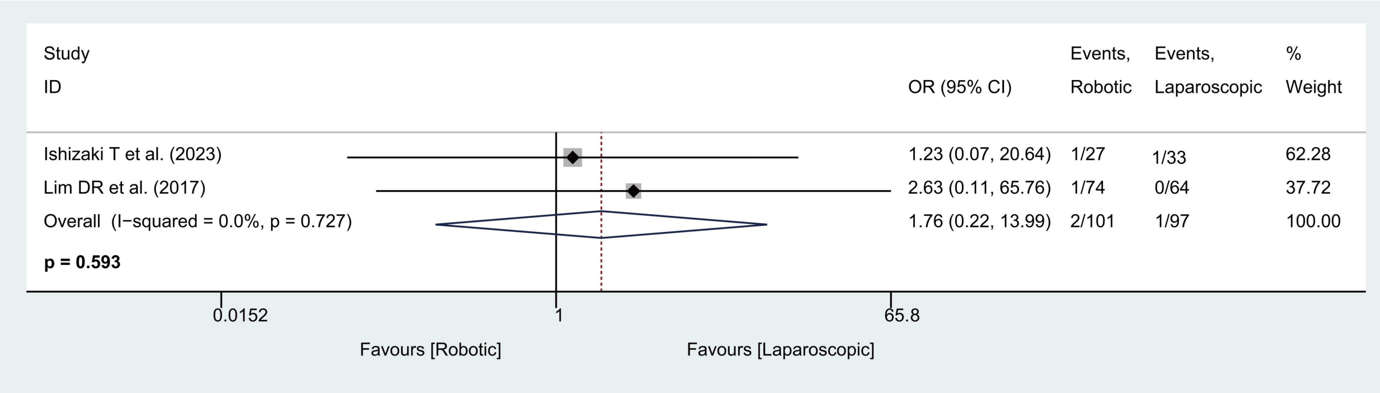

Figure S10. Forest plot of lymphatic leakage in the robotic and laparoscopic groups. Abbreviation: OR, odds ratio; CI, confidence interval.

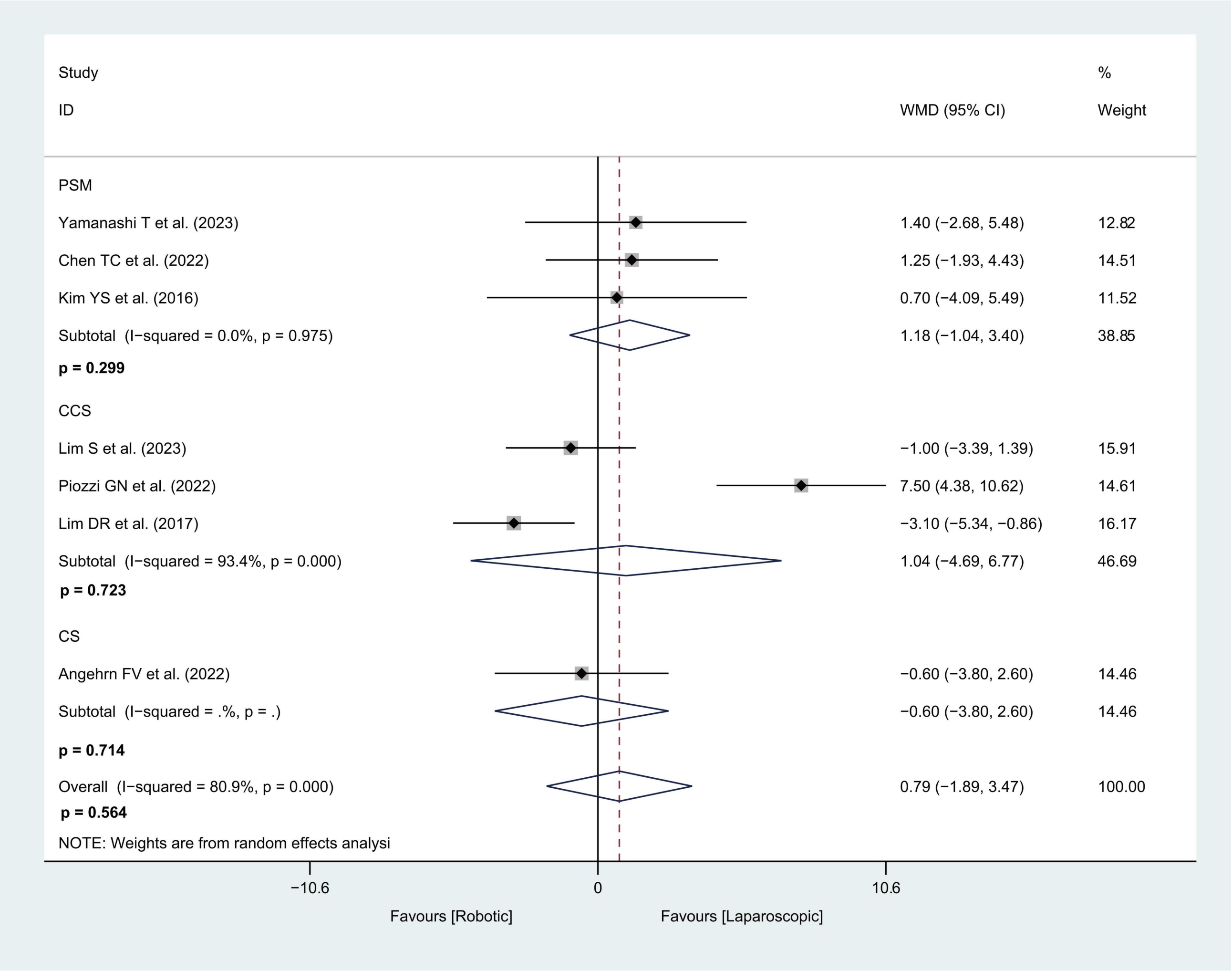

Figure S11. Subgroup analysis results of the total number of lymph nodes harvested stratified by study design. Abbreviation: PSM, propensity score-matched study; CCS, case-control study; CS, cohort study; WMD, weighted mean difference; CI, confidence interval.

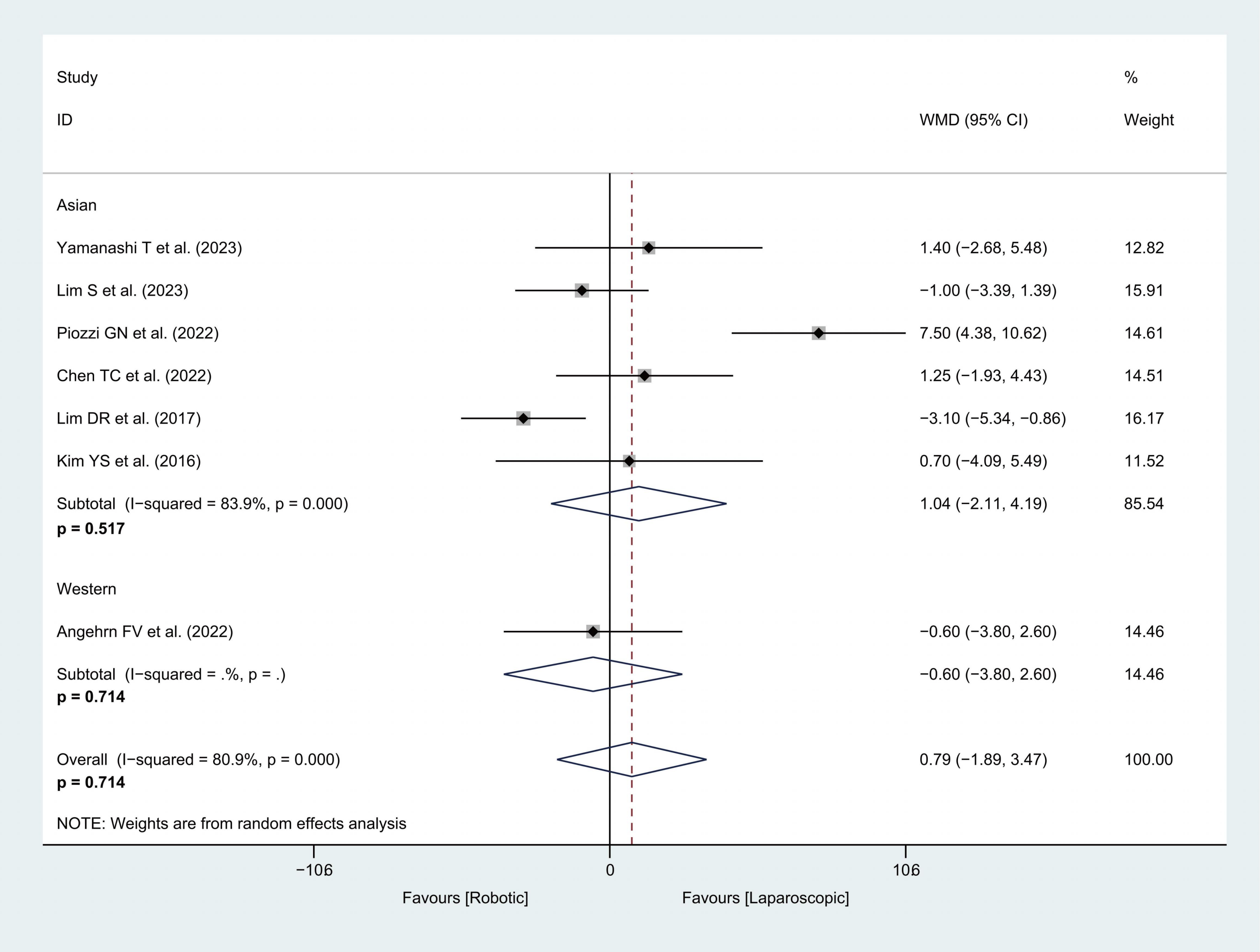

Figure S12. Subgroup analysis results of the total number of lymph nodes harvested stratified by region. Abbreviation: WMD, weighted mean difference; CI, confidence interval.

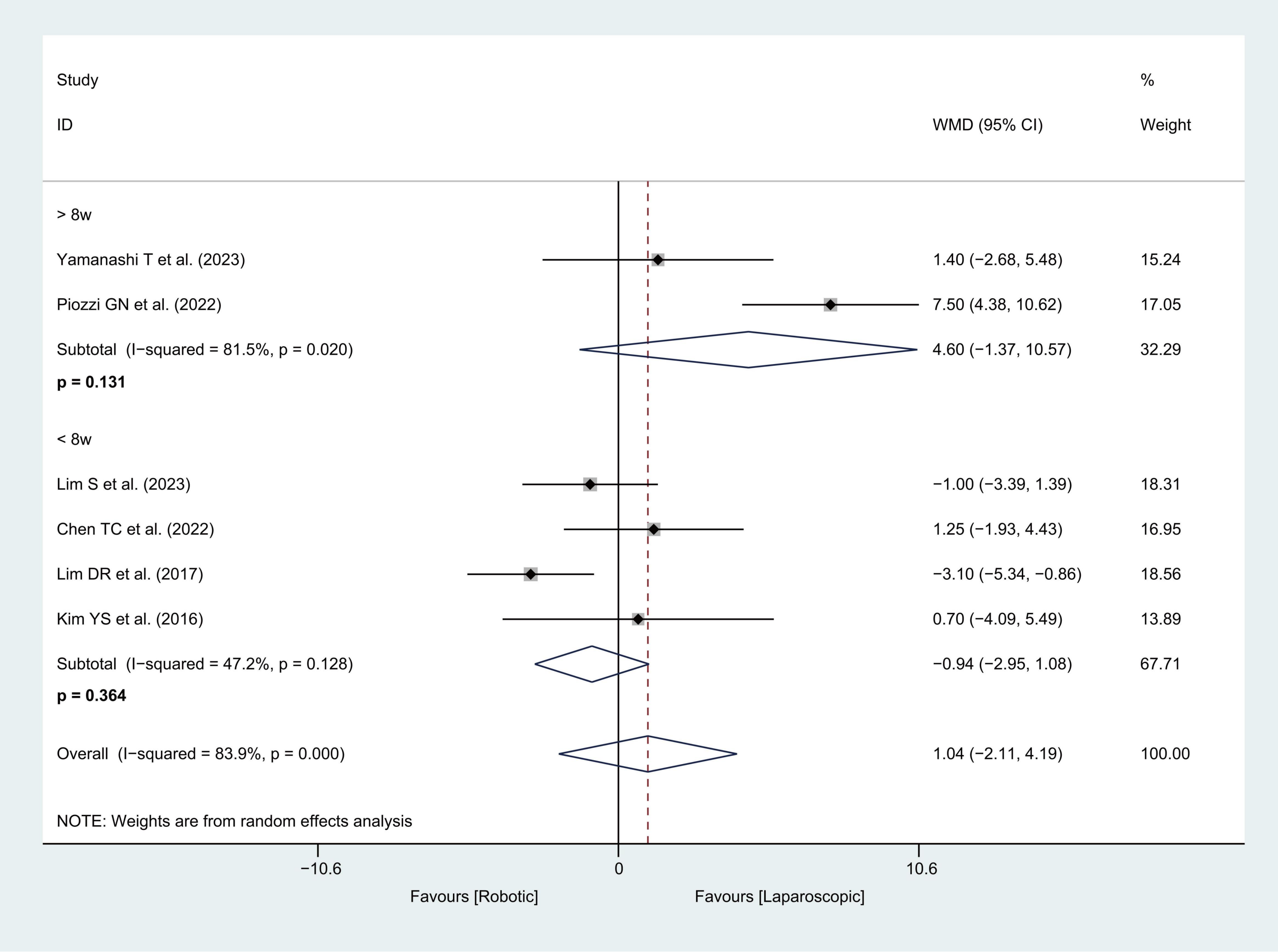

Figure S13. Subgroup analysis results of the total number of lymph nodes harvested stratified by timing of surgery. Abbreviation: WMD, weighted mean difference; CI, confidence interval.

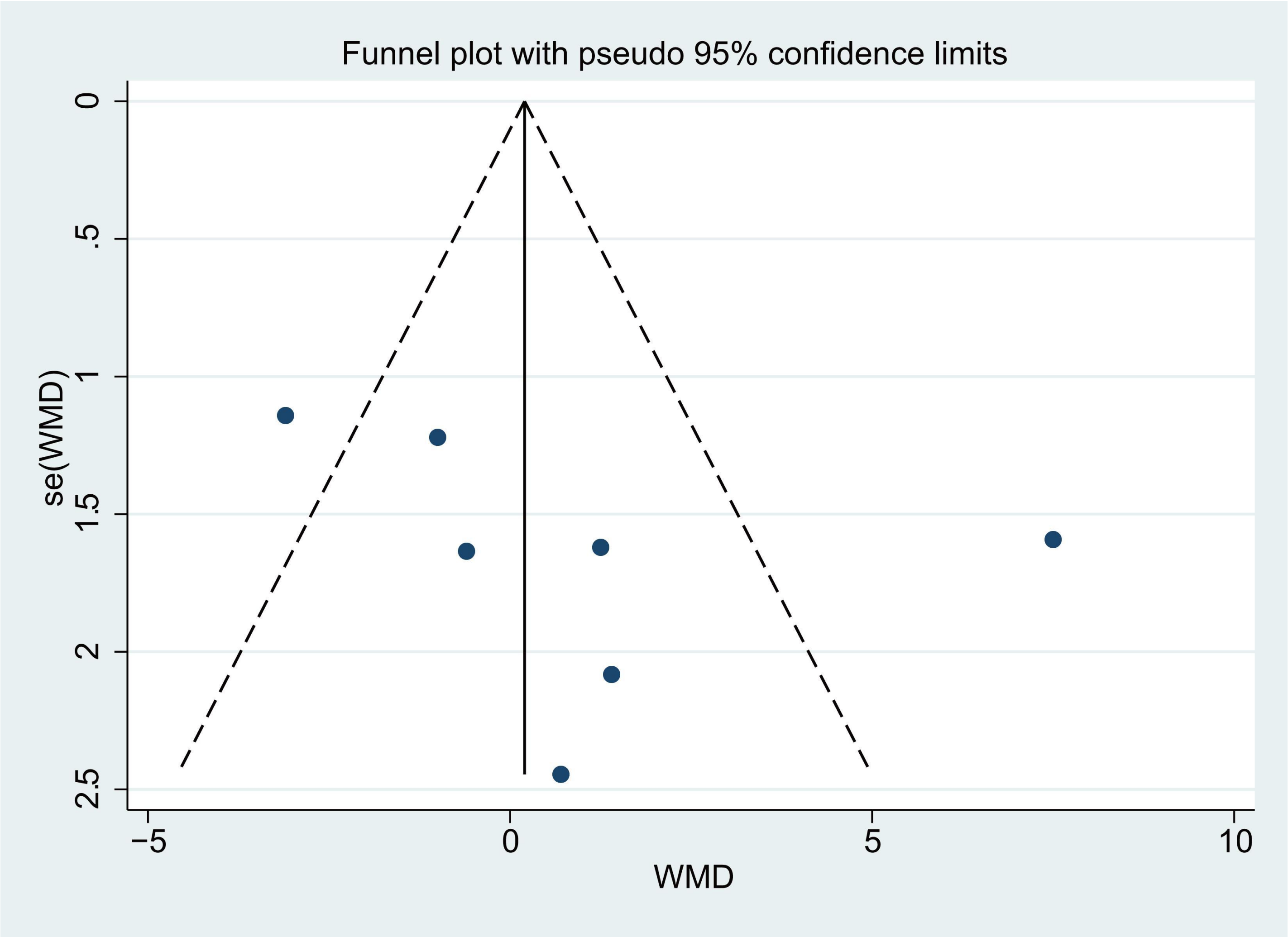

Figure S14. Funnel plot for publication bias of the total number of lymph nodes harvested.

Abbreviation: WMD, weighted mean difference.

A

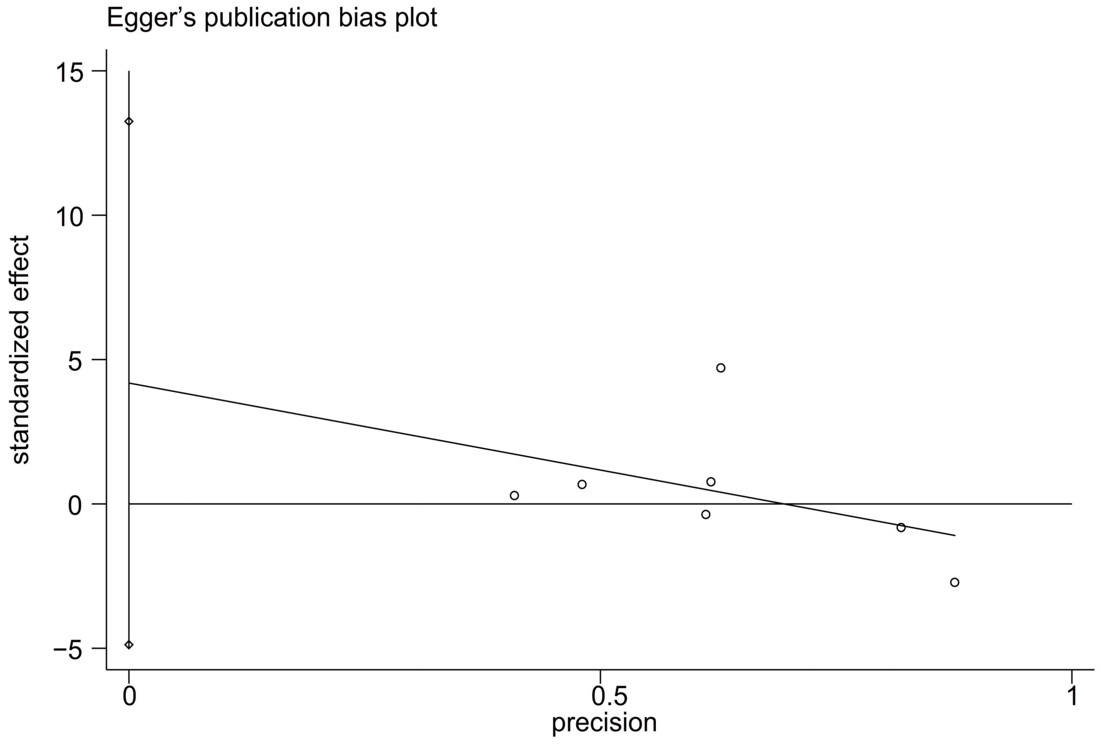

p = 0.288

B

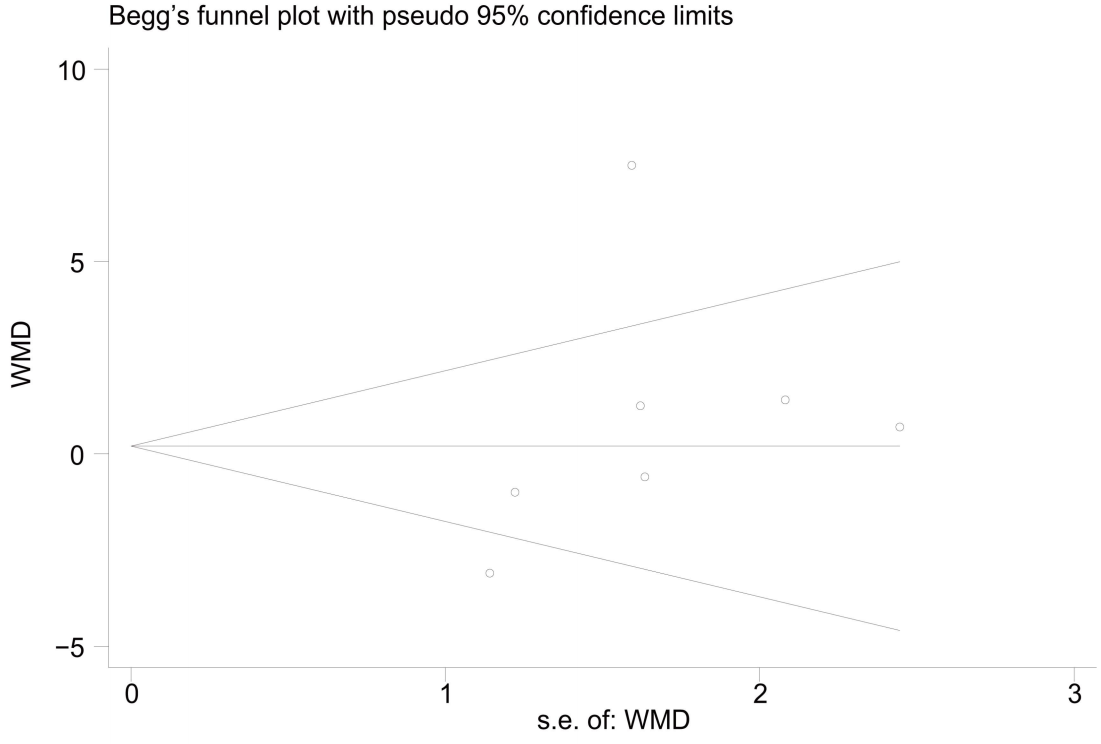

p = 0.548

Figure S15. Plots of publication bias of the total number of lymph nodes harvested. Fig. A. Egger's publication bias plot (P = 0.288); Fig. B. Begg's funnel plot (P = 0.548). Abbreviation: WMD, weighted mean difference.

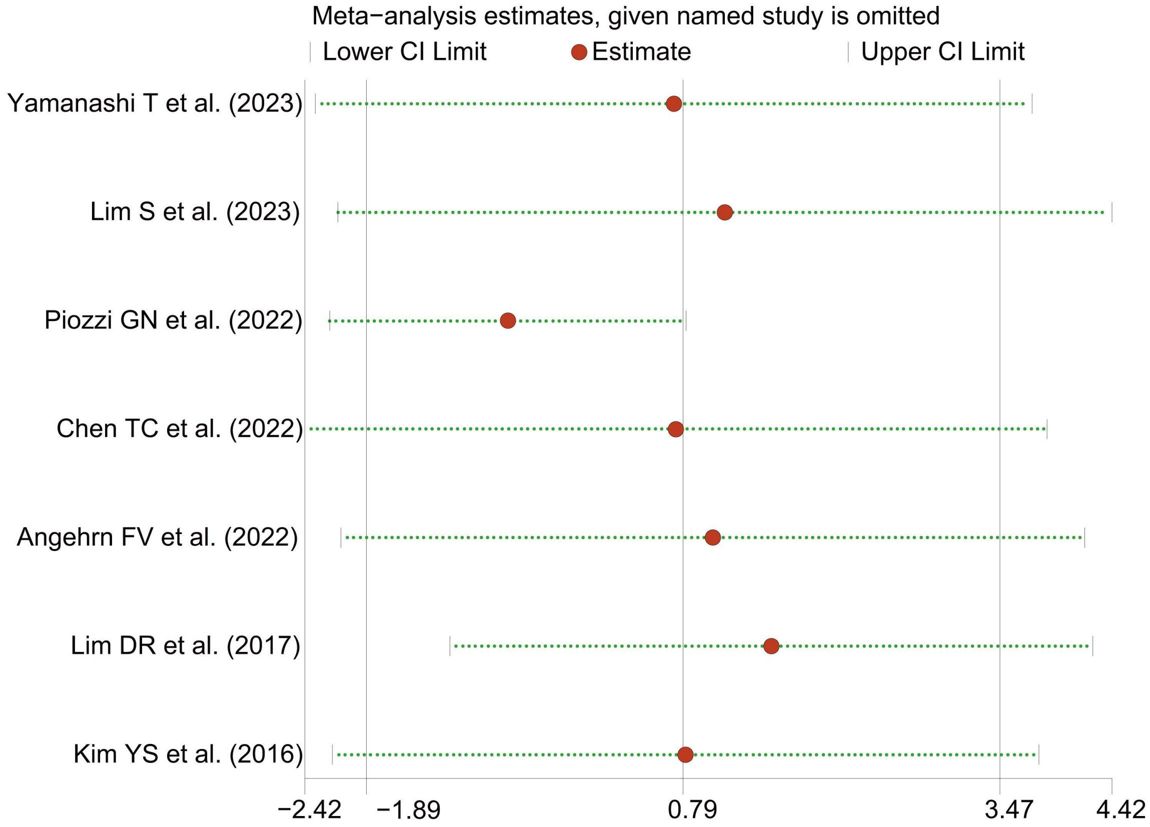

Figure S16. Sensitivity analysis of the total number of lymph nodes harvested.

Abbreviation: CI, confidence interval.

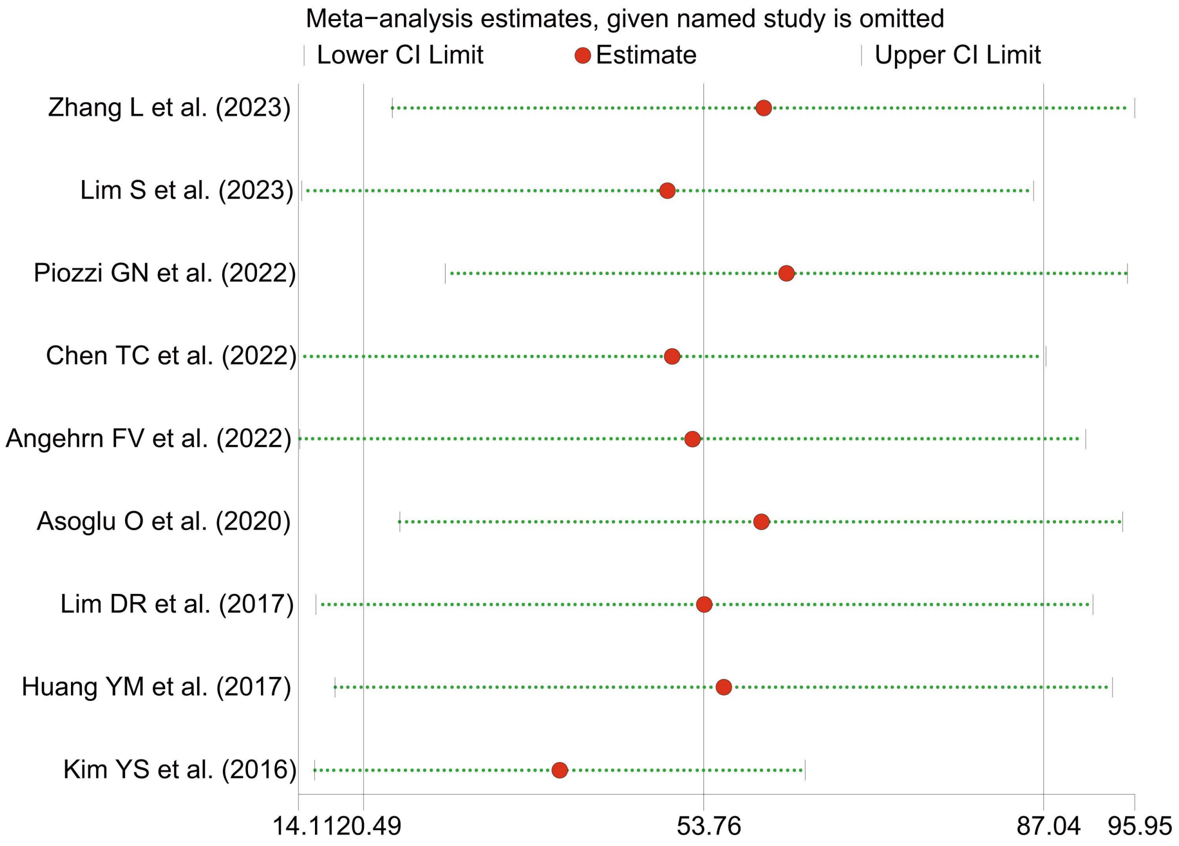

Figure S17. Sensitivity analysis of the operation time. Abbreviation: CI, confidence interval.

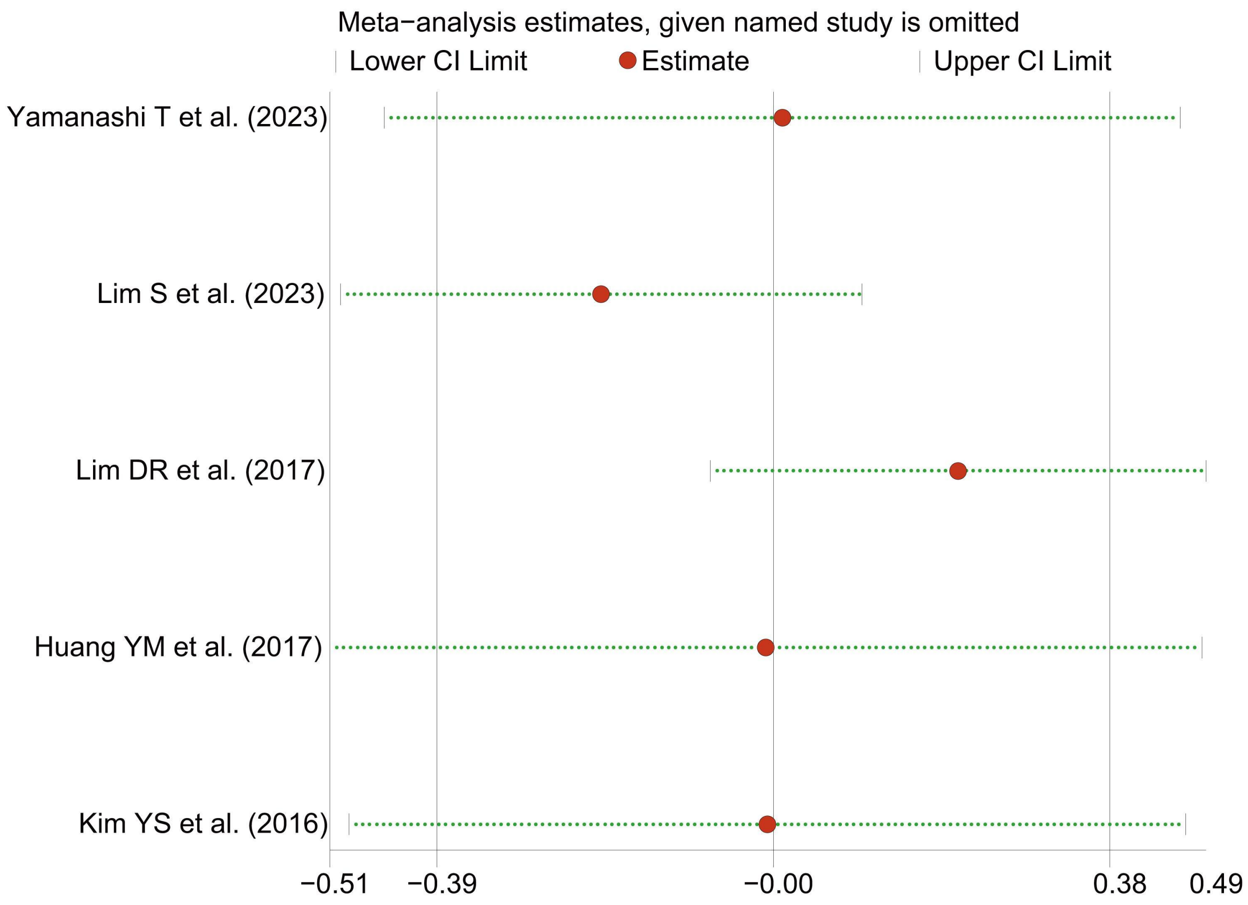

Figure S18. Sensitivity analysis of the distal resection margin. Abbreviation: CI, confidence interval.
